# Functional Cross-Talk of MbtH-Like Proteins During Thaxtomin Biosynthesis in the Potato Common Scab Pathogen *Streptomyces scabiei*

**DOI:** 10.3389/fmicb.2020.585456

**Published:** 2020-10-15

**Authors:** Yuting Li, Kapil Tahlan, Dawn R.D. Bignell

**Affiliations:** Department of Biology, Memorial University of Newfoundland, St. John’s, NL, Canada

**Keywords:** *Streptomyces*, plant pathogen, specialized metabolism, non-ribosomal peptides, thaxtomin, phytotoxin

## Abstract

Thaxtomin A is a potent phytotoxin that serves as the principle pathogenicity determinant of the common scab pathogen, *Streptomyces scabiei*, and is also a promising natural herbicide for agricultural applications. The biosynthesis of thaxtomin A involves the non-ribosomal peptide synthetases (NRPSs) TxtA and TxtB, and an MbtH-like protein (MLP), TxtH, which may function as a chaperone by promoting the proper folding of the two NRPS enzymes in *S. scabiei*. MLPs are required for the proper function of many NRPS enzymes in bacteria, and they are often capable of interacting with NRPSs from different biosynthetic pathways, though the mechanism by which this occurs is still poorly understood. To gain additional insights into MLP functional cross-talk, we conducted a broad survey of MLPs from diverse phylogenetic lineages to determine if they could functionally replace TxtH. The MLPs were assessed using a protein solubility assay to determine whether they could promote the soluble expression of the TxtA and TxtB adenylation domains. In addition, the MLPs were tested for their ability to restore thaxtomin production in a *S. scabiei* mutant that lacked TxtH and other endogenous MLPs. Our results showed that the MLPs investigated vary in their ability to exhibit functional cross-talk with TxtH, with two of the MLPs being unable to compensate for the loss of TxtH in the assays performed. The ability of an MLP to serve as a functional partner for the thaxtomin NRPS was not correlated with its overall amino acid similarity with TxtH, but instead with the presence of highly conserved residues. *In silico* structural analysis of TxtH in association with the TxtA and TxtB adenylation domains revealed that several such residues are situated at the predicted interaction interface, suggesting that they might be critical for promoting functional interactions between MLPs and the thaxtomin NRPS enzymes. Overall, our study provides additional insights into the mechanism of MLP cross-talk, and it enhances our understanding of the thaxtomin biosynthetic machinery. It is anticipated that our findings will have useful applications for both the control of common scab disease and the commercial production of thaxtomin A for agricultural use.

## Introduction

Non-ribosomal peptides (NRPs) are a major class of specialized metabolites produced by certain bacteria and filamentous fungi (Marahiel et al., 1997). The biosynthesis of NRPs is performed by non-ribosomal peptide synthetases (NRPSs), which are large multienzyme complexes composed of modules that are each responsible for the incorporation of an amino acid into the growing peptide (Finking and Marahiel, 2004; Strieker et al., 2010). Each module typically constitutes three core domains: an adenylation (A-) domain, a peptidyl carrier protein (PCP-) domain and a condensation (C-) domain. The A-domain selects a preferred amino acid substrate to initiate the adenylation reaction using Mg⋅ATP. The activated amino acyl-AMP intermediate is then covalently tethered to the downstream PCP-domain, which serves as the transport unit enabling the bound substrate to move between the different catalytic centers. The C-domain catalyzes the amide bond formation between adjacent PCP-bound intermediates. The biosynthesis of NRPs can involve additional domains that either incorporate modifications into the product or release it from the assembly line (Finking and Marahiel, 2004; Hur et al., 2012; Süssmuth and Mainz, 2017). Furthermore, some NRPSs require auxiliary proteins, including members of the MbtH-like protein (MLP) superfamily, for the optimal activity (Baltz, 2011).

MbtH-like proteins are named after the MbtH protein, which is an integral component in the biosynthesis of the siderophore mycobactin in *Mycobacterium tuberculosis* (Quadri et al., 1998; McMahon et al., 2012). Proteins belonging to this family are generally small in size (approximately 60–70 amino acids) and are often found within NRP biosynthetic gene clusters (BGCs) that produce antibiotics or siderophores (Baltz, 2011). Several studies have demonstrated a role for these proteins as chaperones in the NRPS assembly line. In these reports, the soluble production of one or more NRPS A-domains in *Escherichia coli* was shown to be reduced or abolished in the absence of the MLP that is from the same biosynthetic pathway as the NRPS, suggesting that the MLP (called the cognate MLP) is required for the proper folding of the A-domain protein (Imker et al., 2010; Boll et al., 2011; McMahon et al., 2012; Zolova and Garneau-Tsodikova, 2012, 2014; Kaniusaite et al., 2020). Additionally, some MLPs have been shown to influence amino acid activation by the corresponding NRPS. In these instances, the NRPS can be heterologously overexpressed in *E. coli* in soluble form in the absence of the cognate MLP, but the purified protein exhibits low or no activity for the target amino acid *in vitro* unless the purified cognate MLP is added to the reaction, or the MLP is co-expressed with the NRPS (Heemstra et al., 2009; Felnagle et al., 2010; Zhang et al., 2010; Boll et al., 2011; Davidsen et al., 2013; Al-Mestarihi et al., 2014; Miller et al., 2016; Schomer et al., 2018). Previous investigations also noted a 1:1 molar stoichiometry of the MLP-A-domain complex for optimal adenylation activity (Felnagle et al., 2010; Zhang et al., 2010; Boll et al., 2011; Davidsen et al., 2013).

Several studies have reported that in bacteria containing multiple MLPs, the production of a particular NRP is only abolished in some cases when all of the MLP homologues are eliminated (Lautru et al., 2007; Wolpert et al., 2007). This suggests that MLPs from different NRP pathways can sometimes functionally replace one another, though the reason for this is currently not clear. In addition, some MLPs from other biosynthetic pathways (referred to as non-cognate MLPs) have been shown to be comparable or sometimes even more efficient in enhancing the solubility and/or adenylation activity of NRPS enzymes as compared to the cognate MLP (Boll et al., 2011; Mori et al., 2018a). For example, the *E. coli* enterobactin (ENT) biosynthetic pathway was used as a model to investigate the ability of different non-cognate MLPs to influence the function of the EntF NRPS in the absence of the cognate MLP, YbdZ. They found that non-cognate MLPs vary in their ability to compensate for the loss of YbdZ in the different assays performed, and that the interactions between MLPs and NRPSs are multifaceted and more complex than previously realized (Schomer and Thomas, 2017).

Recently, we examined the importance of MLPs in the biosynthesis of thaxtomin A, which is the principle pathogenicity determinant of the potato common scab pathogen *Streptomyces scabiei* (syn. *S. scabies*). Thaxtomin A is a novel nitrated 2,5-diketopiperazine that exhibits potent phytotoxicity against both monocot and dicot plants (King et al., 2001), and it is considered a promising bioherbicide for the control of weed growth (Leep et al., 2010; Koivunen and Marrone, 2013). Production of thaxtomin A in *S. scabiei* is mediated by a BGC that includes two NRPS-encoding genes, *txtA* and *txtB*, which generate the *N-*methylated cyclic dipeptide backbone, and a P450 monooxygenase-encoding gene, *txtC*, which is responsible for the post-cyclization hydroxylation steps (reviewed in Li et al., 2019b). Both TxtA and TxtB contain the three core domains (A-PCP-C) together with a methylation domain integrated into the C-terminal region of the A-domain (Huguet-Tapia et al., 2016). The arrangement of the core domains is unusual when compared to most other NRPSs, which typically have a C-A-PCP domain arrangement (Süssmuth and Mainz, 2017). Immediately downstream of *txtB* is the *txtH* gene, which encodes an MLP that is required for the soluble expression of the TxtA and TxtB A-domains (referred to herein as TxtA^*A*^ and TxtB^*A*^) in *E. coli*, suggesting that it exhibits a chaperone function in *S. scabiei* (Li et al., 2019a). Deletion of *txtH* in *S. scabiei* significantly reduced thaxtomin A production levels, though some production could still occur. In contrast, production was completely abolished when two non-cognate MLP-encoding genes (*mlp*_*lipo*_ and *mlp*_*scab*_) located elsewhere on the chromosome were also deleted. The production of thaxtomin A in the MLP triple mutant could be restored by overexpression of *txtH, mlp_*lipo*_*, or *mlp*_*scab*_, while overexpression of two non-cognate MLPs from other *Streptomyces* species failed to do so (Li et al., 2019a). Overall, our results showed that the TxtH MLP plays a key role in the biosynthesis of thaxtomin A, and that some but not all non-cognate MLPs can functionally replace TxtH in the thaxtomin biosynthetic pathway.

In this study, we aimed to further investigate the mechanism of MLP cross-talk by examining the ability of various MLPs from different bacterial species to functionally replace TxtH during the biosynthesis of thaxtomin A. Using protein expression analysis in *E. coli* combined with thaxtomin A production assays in *S. scabiei*, we show that the different MLPs vary in their ability to exhibit functional overlaps with TxtH. Additionally, we conducted an *in silico* structural analysis of the protein complex involving the thaxtomin (Txt) A-domains with TxtH in order to identify potential residues that may play a key role in the Txt MLP-NRPS interaction. Our work not only provides additional insights into the mechanism of MLP functional cross-talk, but it also enhances our understanding of the thaxtomin biosynthetic machinery, and this in turn could have useful applications for both the control of common scab disease and the commercial production of thaxtomin A for agricultural use.

## Materials and Methods

### Bacterial Strains, Culture Conditions and Maintenance

*Escherichia coli* strains used in this study are listed in Table 1. Strains were routinely cultivated at 37°C unless otherwise indicated. Liquid cultures were grown with shaking (200–250 rpm) in Luria-Bertani (LB) Lennox medium (Fisher Scientific, Ottawa, ON, Canada), low salt LB broth (1% w/v tryptone; 0.5% w/v yeast extract; and 0.25% w/v NaCl), super optimal broth (SOB) or super optimal broth with catabolite repression (SOC) medium (New England Biolabs, Whitby, ON, Canada), while solid cultures were grown on LB Lennox (or low salt LB) medium containing 1.5% w/v agar (NEOGEN, Michigan, United States). When required, the solid or liquid growth media were supplemented with antibiotics as described before (Li et al., 2019a). *E. coli* strains were maintained at 4°C for short-term storage or at −80°C in 20% v/v glycerol for long-term storage (Sambrook and Russell, 2001).

**TABLE 1:** Bacterial strains used in this study.

Strain	Description	Resistance^†^	References or source
***Escherichia coli* strains**			
DH5α	General cloning host	n/a	Gibco-BRL
NEB5α	DH5α derivative, high efficiency competent cells	n/a	New England Biolabs
BL21(DE3)	Source of genomic DNA for amplifying the *ybdZ* coding sequence	n/a	New England Biolabs
BL21(DE3)*ybdZ:aac(3)IV*	BL21(DE3) derivative, *ybdZ* replaced with an apramycin resistance cassette [*aac(3)IV]*	Apra^*R*^	Herbst et al., 2013
ET12567/pUZ8002	*dam*^–^, *dcm*^–^, *hsdS*^–^; non-methylating conjugation host	Kan^*R*^, Cml^*R*^	Kieser et al., 2000
***Streptomyces* strains**			
*Streptomyces scabiei* 87.22	Wild-type strain	n/a	Loria et al., 1995
*S. scabiei* Δ*txtH*	87.22 derivative in which the *txtH* MLP-coding gene has been deleted	Apra^*R*^	Li et al., 2019a
*S. scabiei* Δ*mlp_*lipo*_/*Δ*txtH/*Δ*mlp*_*scab*_	*S. scabiei* 87.22 derivative in which the *SCAB3331*(*mlp_*lipo*_), txtH* and *SCAB85461*(*mlp_*scab*_)* MLP-coding genes have been deleted	Apra^*R*^, Hyg^*R*^	Li et al., 2019a
*Streptomyces coelicolor* A3(2) M145	Source of genomic DNA for amplifying the *cdaX* and *cchK* coding sequences	n/a	Kieser et al., 2000
*Streptomyces* sp. 11-1-2	Source of genomic DNA for amplifying the *CGL27_RS10110* and *CGL27_RS02360* coding sequences	n/a	Bown and Bignell, 2017
*Streptomyces europaeiscabiei* 89-04	Source of genomic DNA for amplifying the *AWZ11_RS05060* coding sequence	n/a	Zhang et al., 2016
*Streptomyces clavuligerus* ATCC27064	Wild-type strain	n/a	ATCC

*^†^Apra^*R*^, Kan^*R*^, Cml^*R*^, and Hyg^*R*^ = apramycin, kanamycin, chloramphenicol and hygromycin resistance, respectively. n/a = not applicable.*

*Streptomyces* strains used in this study are listed in Table 1. Strains were routinely cultured at 28°C unless otherwise indicated. Liquid cultures were typically grown with shaking (200 rpm) in trypticase soy broth (TSB; BD Biosciences, Mississauga, ON, Canada) medium with stainless steel springs. *S. scabiei* cultures for analysis of thaxtomin production were prepared by inoculating oat bran broth containing 0.35% w/v cellobiose (OBBC) with TSB seed cultures of each strain and then incubating at 25°C for 7 days as described before (Li et al., 2019a). Plate cultures were grown on potato mash agar (PMA; Fyans et al., 2016), International *Streptomyces* Project Medium 4 (ISP-4; BD Biosciences), nutrient agar (BD Biosciences, 1.5% w/v agar), and soy flour mannitol agar (SFMA; Kieser et al., 2000). When required, the growth medium was supplemented with apramycin, nalidixic acid, kanamycin or hygromycin B (50 μg/ml final concentration; Millipore Sigma, Oakville, ON, Canada).

### Plasmids, Primers and DNA Manipulation

Plasmids used in this study are listed in Table 2. Standard molecular biology procedures were implemented for all DNA manipulations performed (Sambrook and Russell, 2001). *Streptomyces* genomic DNA was isolated from mycelia harvested from TSB cultures using the DNeasy Blood & Tissue Kit as per the manufacturer’s protocol (QIAgen Inc, Canada). The nucleotide sequences of the MLP-encoding genes *MXAN_3118* (from *Myxococcus xanthus* DK1622), *RHA1_ro04717* (from *Rhodococcus jostii* RHA1), *PA2412* (from *Pseudomonas aeruginosa* PA01), and *ybdZ* [from *E. coli* BL21(DE3)] were codon optimized for expression in *Streptomyces* using a webserver^1^ from Integrated DNA Technologies (Coralville, IA, United States). The codon optimized sequences along with *cloY* (from *Streptomyces roseochromogenes* subsp. *oscitans* DS12.976) and *comB* (from *Streptomyces lavendulae*) were then synthesized with 30–60 bp flanking regions by TWIST BIOSCIENCE (South San Francisco, CA, United States; Supplementary Data File 1). All oligonucleotide primers used for cloning, PCR and sequencing were purchased from Integrated DNA Technologies and are listed in Supplementary Table 1. Restriction enzymes were purchased from New England Biolabs. PCR was routinely performed using Phusion or *Taq* DNA polymerase (New England Biolabs) according to the manufacturer’s instructions, except that 5% v/v DMSO was included in the reactions. DNA sequencing was performed by The Centre for Applied Genomics (Toronto, ON, Canada).

**TABLE 2:** Plasmids used in this study.

Plasmid	Description	Resistance^†^	References or source
pGEM-T EASY	General cloning vector	Amp^*R*^	Promega Corporation
pGEM-T EASY/*comB*	pGEM-T EASY derivative containing a 312 bp insert of the *comB* gene with flanking regions	Amp^*R*^	This study
pGEM-T EASY/*cloY*	pGEM-T EASY derivative containing a 306 bp insert of the *cloY* gene with flanking regions	Amp^*R*^	This study
pGEM-T EASY/*MXAN_3118*	pGEM-T EASY derivative containing a 306 bp insert of the *MXAN_3118* gene^‡^ with flanking regions	Amp^*R*^	This study
pGEM-T EASY/*PA2412*	pGEM-T EASY derivative containing a 309 bp insert of the *PA2412* gene^‡^ with flanking regions	Amp^*R*^	This study
pGEM-T EASY/*RHA1_ro04717*	pGEM-T EASY derivative containing a 342 bp insert of the *RHA1_ro04717* gene^‡^ with flanking regions	Amp^*R*^	This study
pGEM-T EASY/*ybdZ*	pGEM-T EASY derivative containing a 300 bp insert of the *ybdZ* gene^‡^ with flanking regions	Amp^*R*^	This study
pET28b	N- or C- terminal 6 × histidine fusion tag protein expression vector with T7 promoter and *lac* operator	Kan^*R*^	Novagen
pET28b/HIS_6_-*txtH*	pET28b derivative containing a DNA fragment for expression of the HIS_6_-TxtH protein	Kan^*R*^	Li et al., 2019a
pET28b/HIS_6_-*cdaX*	pET28b derivative containing a DNA fragment for expression of the HIS_6_-CdaX protein	Kan^*R*^	This study
pET28b/HIS_6_-*cchK*	pET28b derivative containing a DNA fragment for expression of the HIS_6_-CchK protein	Kan^*R*^	This study
pET28b/HIS_6_-*SCLAV_p1293*	pET28b derivative containing a DNA fragment for expression of the HIS_6_-SCLAV_p1293 protein	Kan^*R*^	This study
pET28b/HIS_6_-*ybdZ*	pET28b derivative containing a DNA fragment for expression of the HIS_6_-YbdZ protein	Kan^*R*^	This study
pET28b/HIS_6_- *CGL27_RS10110*	pET28b derivative containing a DNA fragment for expression of the HIS_6_-CGL27_RS10110 protein	Kan^*R*^	This study
pET28b/HIS_6_- *CGL27_RS02360*	pET28b derivative containing a DNA fragment for expression of the HIS_6_-CGL27_RS02360 protein	Kan^*R*^	This study
pET28b/HIS_6_- *AWZ11_RS05060*	pET28b derivative containing a DNA fragment for expression of the HIS_6_-AWZ11_RS05060 protein	Kan^*R*^	This study
pET28b/HIS_6_-*comB*	pET28b derivative containing a DNA fragment for expression of the HIS_6_-ComB protein	Kan^*R*^	This study
pET28b/HIS_6_-*cloY*	pET28b derivative containing a DNA fragment for expression of the HIS_6_-CloY protein	Kan^*R*^	This study
pET28b/HIS_6_-*MXAN_3118*	pET28b derivative containing a DNA fragment^‡^ for expression of the HIS_6_-MXAN_3118 protein	Kan^*R*^	This study
pET28b/HIS_6_-*PA2412*	pET28b derivative containing a DNA fragment^‡^ for expression of the HIS_6_-PA2412 protein	Kan^*R*^	This study
pET28b/HIS_6_-*RHA1_ro04717*	pET28b derivative containing a DNA fragment^‡^ for expression of the HIS_6_-RHA1_ro04717 protein	Kan^*R*^	This study
pACYCDuet-1	N-terminal 6 × histidine fusion tag expression vector with T7 promoter and *lac* operator	Cml^*R*^	Novagen
pACYCDuet-1/HIS_6_-*txtA*^*A*^	pACYCDuet-1 derivative containing a DNA fragment for expression of the HIS_6_-TxtA^*A*^ protein	Cml^*R*^	Li et al., 2019a
pACYCDuet-1/HIS_6_-*txtB*^*A*^	pACYCDuet-1 derivative containing a DNA fragment for expression of the HIS_6_-TxtB^*A*^ protein	Cml^*R*^	Li et al., 2019a
pRFSRL16	Harbors the *egfp* gene downstream of the *ermE*p* promoter and an RBS; integrates into the ΦC31 *attB* site	Apra^*R*^, Kan^*R*^	Joshi et al., 2010
pRFSRL16/*txtH*	pRFSRL16 derivative in which *egfp* is replaced with the *S. scabiei txtH* gene	Apra^*R*^, Kan^*R*^	This study
pRFSRL16/*mlp*_*lipo*_	pRFSRL16 derivative in which *egfp* is replaced with the *S. scabiei mlp_*lipo*_* gene	Apra^*R*^, Kan^*R*^	This study
pRFSRL16/*cdaX*	pRFSRL16 derivative in which *egfp* is replaced with the *S. coelicolor cdaX* gene	Apra^*R*^, Kan^*R*^	This study
pRFSRL16/*cchK*	pRFSRL16 derivative in which *egfp* is replaced with the *S. coelicolor cchK* gene	Apra^*R*^, Kan^*R*^	This study
pRFSRL16/*SCLAV_p1293*	pRFSRL16 derivative in which *egfp* is replaced with the *S. clavuligerus SCLAV_p1293* gene	Apra^*R*^, Kan^*R*^	This study
pRFSRL16/*ybdZ*	pRFSRL16 derivative in which *egfp* is replaced with the *E. coli* BL21(DE3) *ybdZ* gene^‡^	Apra^*R*^, Kan^*R*^	This study
pRFSRL16/*CGL27_RS10110*	pRFSRL16 derivative in which *egfp* is replaced with the *Streptomyces* sp. 11-1-2 *CGL27_RS10110* gene	Apra^*R*^, Kan^*R*^	This study
pRFSRL16/*CGL27_RS02360*	pRFSRL16 derivative in which *egfp* is replaced with the *Streptomyces* sp. 11-1-2 *CGL27_RS02360* gene	Apra^*R*^, Kan^*R*^	This study
pRFSRL16/*AWZ11_RS05060*	pRFSRL16 derivative in which *egfp* is replaced with the *S. europaeiscabiei* 89-04 *AWZ11_RS05060* gene	Apra^*R*^, Kan^*R*^	This study
pRFSRL16/*comB*	pRFSRL16 derivative in which *egfp* is replaced with the *S. lavendulae comB* gene	Apra^*R*^, Kan^*R*^	This study
pRFSRL16/*cloY*	pRFSRL16 derivative in which *egfp* is replaced with the *S. roseochromogenes subsp. oscitans* DS12.976 *cloY* gene	Apra^*R*^, Kan^*R*^	This study
pRFSRL16/*MXAN_3118*	pRFSRL16 derivative in which *egfp* is replaced with *M. xanthus* DK1622 *MXAN_3118* gene^‡^	Apra^*R*^, Kan^*R*^	This study
pRFSRL16/*PA2412*	pRFSRL16 derivative in which *egfp* is replaced with the *P. aeruginosa* PA01 *PA2412* gene^‡^	Apra^*R*^, Kan^*R*^	This study
pRFSRL16/*RHA1_ro04717*	pRFSRL16 derivative in which *egfp* is replaced with the *R. jostii* RHA1 *RHA1_ro04717* gene^‡^	Apra^*R*^, Kan^*R*^	This study

*^†^Amp^*R*^, Apra^*R*^, Kan^*R*^, and Cml^*R*^ = ampicillin, apramycin, kanamycin, and chloramphenicol resistance, respectively. ^‡^Gene sequence was codon optimized for expression in *Streptomyces* spp.*

### Construction of *E. coli* Protein Expression Plasmids

Construction of the expression plasmids pACYCDuet-1/HIS_6_-*txtA*^*A*^, pACYCDuet-1/HIS_6_-*txtB*^*A*^, and pET28b/HIS_6_-*txtH* was described in Li et al. (2019a). The MLP-encoding genes *CGL27_RS10110* and *CGL27_RS02360* from *Streptomyces* sp. 11-1-2, *cdaX* and *cchK* gene from *S. coelicolor*, *SCLAV_p1293* from *S. clavuligerus*, *AWZ11_RS05060* from *S. europaeiscabiei*, and *ybdZ* from *E. coli* (Table 1) were PCR-amplified using genomic DNA as template and using primers with *Nde*I and *Eco*RI restriction sites added. The PCR products were purified using the Wizard SV Gel and PCR Clean-Up System (Promega, Canada) and were then digested with *Nde*I and *Eco*RI and ligated into similarly digested pET28b (Table 2). The synthetic gene fragments for *comB*, *cloY*, *MXAN3118*, *PA2412*, and *RHA1_ro04717* were cloned into the pGEM-T EASY vector (Promega North America, United States) as per the manufacturer’s instructions (Table 2). The resulting plasmids were then used as templates for PCR amplification using primers listed in Supplementary Table 1. The gene products were each purified and then cloned into the *Nde*I/*Eco*RI restriction sites of pET28b except for *comB*, which was cloned into the *Nde*I/*Bam*HI vector restriction sites (due to the presence of an *Eco*RI site within the gene sequence). The cloned inserts in all constructed expression vectors were then verified by DNA sequencing.

### Co-Expression of HIS_6_-TxtA^*A*^ and HIS_6_-TxtB^*A*^ With HIS_6_-Tagged MLPs

The co-expression of HIS_6_-TxtA^*A*^ and HIS_6_-TxtB^*A*^ with HIS_6_-tagged MLPs was conducted as previously described (Li et al., 2019a). Briefly, the expression strain *E. coli* BL21(DE3)*ybdZ:aac(3)IV* (Table 1) containing either pACYCDuet-1/HIS_6_-*txtA*^*A*^ or pACYCDuet-1/HIS_6_-*txtB^*A*^*, with and without a pET28b-derived MLP expression plasmid (Table 2), was cultured overnight in 3 mL of LB medium supplemented with 1% w/v glucose and the appropriate antibiotics. The overnight cultures were subcultured into fresh LB medium containing appropriate antibiotics, and the cultures were incubated at 37°C and 200 rpm until the OD_600_ reached 0.4–0.6. The production of the HIS_6_-tagged proteins was induced by adding 1 mM isopropyl β-d-thiogalactopyranoside (IPTG) and then incubating the cultures at 16°C and 200 rpm for 48 h. Cells from 1 mL of culture were harvested and were resuspended in 200 μL of 50 mM Tris–HCl (pH 8.0) containing 1 × cOmplete EDTA-free protease inhibitor. The cells were lysed by sonication and the cell debris was removed by centrifugation. The soluble proteins were collected, and the protein concentration was quantified using a Bradford protein assay kit (Fisher Scientific).

### Western Blot Analysis

Equal amounts (10 μg) of total soluble protein extracts were separated by sodium dodecyl sulfate polyacrylamide gel electrophoresis (SDS-PAGE) on a 15% w/v gel before being transferred to an Amersham^TM^ Hybond^TM^ ECL membrane (GE Healthcare Canada Inc., Canada) as described by the manufacturer’s instructions. To ensure equal loading of each protein sample, separate polyacrylamide gels were prepared and then stained with Coomassie Brilliant Blue stain (50% v/v methanol, 10% v/v glacial acetic acid, and 0.1% w/v Coomassie Blue; Supplementary Figure 1). Membranes were blocked overnight in TBS-T buffer (50 mM Tris–HCl pH 7.6, 150 mM NaCl, and 0.05% v/v Tween 20) containing 5% w/v skim milk, and were then incubated with 6 × His Epitope Tag Antibody (Fisher Scientific) at a 1:2000 dilution. The membranes were washed several times with TBS-T buffer and were then incubated with the secondary antibody (Fisher Scientific) at a 1:2000 dilution. The membranes were processed using the ECL^TM^ western blotting high sensitivity detection reagent (GE Healthcare) and were visualized by ImageQuant LAS4000 Biomolecular Imager (GE Healthcare). The intensity of the HIS_6_-TxtA^*A*^ and HIS_6_-TxtB^*A*^ protein bands was quantified using ImageJ (Schneider et al., 2012) and the average% band intensity relative to the appropriate control (HIS_6_-TxtA^*A*^ or HIS_6_-TxtB^*A*^ co-expressed with HIS_6_-TxtH) was calculated from triplicate membranes (Supplementary Figure 2) that were prepared using protein extracts from three independent cultures for each strain. Statistical analysis of the results was conducted in Minitab 19 (Minitab LLC, State College, PA, United States) using one-way ANOVAs with *a posteriori* multiple comparisons of least squared means performed using the Tukey test. *P* values ≤ 0.05 were considered as statistically significant in all analyses.

### Construction of Plasmids for Overexpression of MLPs in *S. scabiei*

The MLP-encoding genes were PCR-amplified using the corresponding pET28b plasmid clone (for *txtH, mlp_*lipo*_, cdaX, cchK, SCLAV_p1293, CGL_RS10110, CGL27_RS02360*, and *AWZ11_RS05060*) or the pGEM-T EASY clone (for *comB, cloY, MXAN3118, PA2412, RHA1_ro04717*, and *ybdZ*) as template (Table 2) and using gene-specific primers (Supplementary Table 1) with *Nde*I and *Not*I restriction sites added. The PCR products were digested with *Nde*I and *Not*I and then ligated into similarly digested pRFSRL16 (Joshi et al., 2010). The resulting plasmids (Table 2) contained the cloned MLP-encoding gene in place of the *egfp* gene in pRFSRL16, and each were verified by sequencing. The plasmids along with the control vector (pRFSRL16) were then introduced into the *S. scabiei*Δ*mlp*_*lipo*_/Δ*txtH*/Δ*mlp*_*scab*_ mutant (Table 1) by intergeneric conjugation with *E. coli* as described before (Kieser et al., 2000).

### Analysis of Thaxtomin Production

Thaxtomins were extracted from *S. scabiei* OBBC cultures and were detected by reverse phase HPLC as described before (Li et al., 2019a). Briefly, each strain was cultured in triplicate, and in the case of the MLP overexpression strains, two different isolates per strain were cultured in triplicate for a total of six cultures. Culture extracts were prepared by extracting the culture supernatants with ethyl acetate, drying the extracts by evaporation, and resuspending the residual material in 100% v/v HPLC-grade methanol. The extracts were analyzed using an Agilent 1260 Infinity Quaternary LC system (Agilent Technologies Canada Inc.) with a Poroshell 120 EC-C18 column (4.6 × 50 mm, 2.7 μm particle size; Agilent Technologies Canada, Inc.) held at a constant temperature of 40°C. An isocratic mobile phase consisting of 30% acetonitrile and 70% water at a constant flow rate of 1.0 mL/min was used for metabolite separation, and metabolites were monitored using a detection wavelength of 380 nm. The normalized total thaxtomin production level for each culture was determined by summing the measured peak area for thaxtomin A, thaxtomin B, and thaxtomin D and then dividing the total area by the measured dry cell weight of the culture. The results for each strain were then averaged among the replicate samples and were reported as the percent thaxtomin production relative to wild-type *S. scabiei* 87.22. Statistical analysis of the results was conducted in Minitab 19 using one-way ANOVAs with *a posteriori* multiple comparisons of least squared means performed using the Tukey test. *P* values ≤ 0.05 were denoted as statistically significant in all analyses.

### Bioinformatics Analysis and Structural Modeling

Identification of the adenylation domain within the TxtA and TxtB amino acid sequences was performed as described previously (Li et al., 2019a). The homologues of TxtH were identified using the NCBI Protein Basic Local Alignment Search Tool (BLASTP)^2^. The cutoff used to select MLPs for analysis was 39% end to end amino acid identity with the *S. scabiei* TxtH. In total, 133 MLPs were chosen from different phyla, and the accession numbers for the proteins used are listed in Supplementary Table 2. Amino acid sequence alignments were generated using ClustalW within the Geneious version 6.1.2 software (Biomatters Ltd.). Phylogenetic trees were constructed from the alignments using the maximum likelihood method in the MEGA X software (Kumar et al., 2018) and using the Whelan and Goldman plus gamma (WAG + G) substitution model (Whelan and Goldman, 2001). Bootstrap analyses were performed with 1000 replicates and the Interactive Tree of Life (iTOL) was used to visualize the tree (Letunic and Bork, 2007)^3^.

The *in silico* 3-dimentional structures of the *S. scabiei* TxtA^*A*^, TxtB^*A*^, and TxtH were prepared using SWISS-MODEL (Biasini et al., 2014). The crystal structure of the TioS NRPS (PDB ID: 5wmm_1; Mori et al., 2018b) from *Micromonospora* sp. ML1 was used as the template for both the TxtA^*A*^ and TxtB^*A*^ models. The model of TxtH was generated based on the crystal structure of the FscK MLP from *Thermobifida fusca* (PDB ID: 6ea3_1; Bruner and Zagulyaeva, unpublished). The generated models (Supplementary Datas 2, 3, 4) were evaluated by different parameters using the SWISS-MODEL webserver (Supplementary Table 3)^4^ and were visualized using PyMOL (DeLano, 2002). The interface between the TioT-TioS complex (PDB ID: 5wmm) and the FscK-FscH complex (PDB ID: 6ea3) was analyzed using the Proteins, Interfaces, Structures, Assemblies software (PISA) server (Krissinel and Henrick, 2007)^5^ for use in homology modeling analysis. The TxtH model was docked with the TxtA^*A*^ or TxtB^*A*^ model in PyMOL based on the location of TioT in the TioT-TioS complex.

## Results and Discussion

### Selection of Non-cognate MLPs for Functional Studies

**FIGURE 1:**
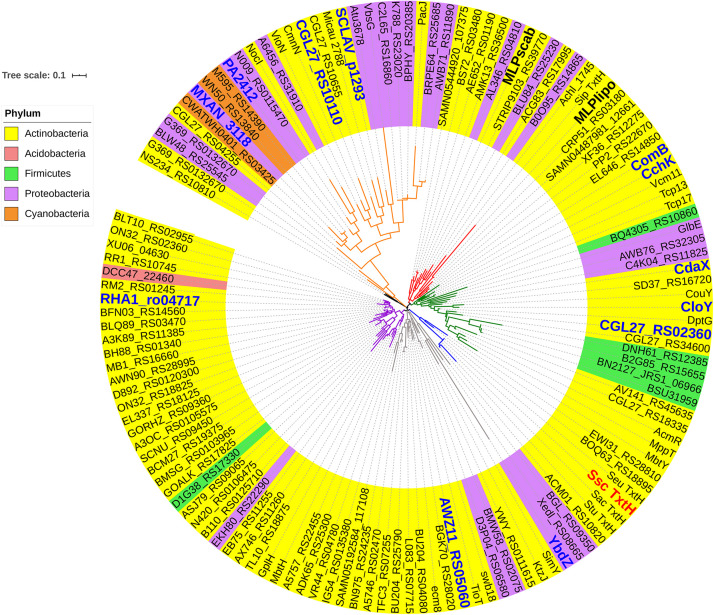
Phylogenetic analysis of MLPs. The phylogeny was generated from the amino acid sequences of 133 MLPs from the database. The MLPs originate from the phyla Actinobacteria (yellow), Acidobacteria (fuchsia), Proteobacteria (purple), Firmicutes (green), and Cyanobacteria (orange). TxtH from *S. scabiei* is highlighted in red, and the two other MLPs encoded in the *S. scabiei* genome (MLP_*lipo*_ and MLP_*scab*_) are indicated in bold. MLPs that were chosen for functional analysis in this study are labeled in blue. Diverse lineages are shown in different colors, and the scale bar indicates the number of amino acid substitutions per site. Information about each MLP is provided in Supplementary Table 2. Ssc, *Streptomyces scabiei*; Sac, *Streptomyces acidiscabies*; Stu, *Streptomyces turgidiscabies*; and Seu, *Streptomyces europaeiscabiei*.

In order to investigate the ability of different non-cognate MLPs to functionally replace TxtH in the thaxtomin biosynthetic pathway, we first conducted a phylogenetic analysis of 133 MLPs from the database, which included TxtH homologues from known or predicted thaxtomin producers, and other previously studied MLPs (Figure 1). This led to the identification of 12 candidate MLPs from diverse phylogenetic clades (Figure 1) that exhibited between 39–59% amino acid identity with TxtH (Table 3). Three of the MLPs originate from different species within the Proteobacteria, while the remaining nine MLPs originate from Actinobacteria, including different species of *Streptomyces* and a strain of *R. jostii* (Figure 1). Eleven of the MLPs are associated with BGCs that are known or predicted to produce different types of NRP metabolites (Table 3), and six are encoded immediately next to a NRPS-encoding gene within BGCs (Supplementary Figure 3). Only one MLP is not encoded within a specific gene cluster and is therefore considered an orphan MLP (Table 3).

**TABLE 3:** Overview of non-cognate MLPs tested in this study and their amino acid sequence identity/similarity to *S. scabiei* TxtH.

Bacterial strain	MLP	Product	Product Class	Identity/similarity to TxtH (%)
*Streptomyces coelicolor* A3(2)	CdaX	Calcium-dependent antibiotic	Cyclic lipodepsipeptide	55/73
*Streptomyces clavuligerus* ATCC 27064	SCLAV_p1293	Putative maduropeptin	NRPS, T1PKS, ectoine, phosphoglycolipid	40/61
*Streptomyces* sp. 11-1-2	CGL27_RS02360	Putative skyllamycin	NRPS, arylpolyene, ladderane	52/73
*Streptomyces* sp. 11-1-2	CGL27_RS10110	Putative toyocamycin	NRPS, nucleoside	41/62
*Escherichia coli* BL21(DE3)	YbdZ	Enterobactin	Siderophore	40/60
*Streptomyces europaeiscabiei* 89-04	AWZ11_RS05060	Putative thiocoraline	NRPS, terpene	57/80
*Streptomyces coelicolor* A3(2)	CchK	Coelichelin	Peptide siderophore	54/70
*Myxococcus xanthus* DK 1622	MXAN_3118	^†^	^†^	39/59
*Pseudomonas aeruginosa* PAO1	PA2412	Pyoverdine	Siderophore	46/63
*Rhodococcus jostii* RHA1	RHA1_ro04717	Putative erythrochelin	NRPS	59/77
*Streptomyces roseochromogenes* subsp. oscitans DS12.976	CloY	Clorobiocin	Aminocoumarin	48/70
*Streptomyces lavendulae*	ComB	Complestatin	Glycopeptide-like	54/71

*^†^MXAN_3118 is not associated with a specific NRP biosynthetic gene cluster.*

Among the chosen MLPs candidates, the importance of several in NRP biosynthesis has been demonstrated in previous studies. For instance, CdaX is encoded by a gene from the known calcium-dependent peptide antibiotic (CDA) BGC in *S. coelicolor* (Table 3) and can functionally replace CchK, which is encoded in the gene cluster responsible for producing the siderophore coelichelin in the same organism. The deletion of either *cdaX* or *cchK* reduces but does not abolish the production of the respective NRP products, while the disruption of both genes completely eliminates the production of both metabolites (Lautru et al., 2007). Additionally, CdaX has been shown to stimulate the activities of L-tyrosine-adenylating enzymes from different NRP biosynthetic pathways (Boll et al., 2011). In contrast, results from our previous study suggested that CdaX is unable to functionally replace TxtH in the thaxtomin biosynthetic pathway (Li et al., 2019a). CloY from the clorobiocin BGC of *S. roseochromogenes* (Table 3; Pojer et al., 2002) is essential for production of the aminocoumarin antibiotic (Wolpert et al., 2007), as it is required for the solubility and adenylation activity of its corresponding NRPS partner, CloH (Boll et al., 2011). The ComB-encoding gene is situated within a glycopeptide-like complestatin NRP BGC from *S. lavendulae* (Chiu et al., 2001) and was recently shown to stimulate the production of several NRPs in the mold *Penicillium chrysogenum*, which does not harbor any MLP-encoding genes in its genome (Zwahlen et al., 2019). YbdZ has been extensively investigated in recent studies and is required for the biosynthesis of the ENT siderophore in *E. coli* (Schomer and Thomas, 2017; Schomer et al., 2018). The deletion of *ybdZ* abolishes ENT production even though its NRPS partner (EntF) is not dependent on the presence of YbdZ for soluble protein production, and biochemical analyses have shown that the solubility and catalytic activity of EntF is significantly enhanced by YbdZ (Felnagle et al., 2010). PA2412 is the MLP associated with the biosynthesis of the siderophore pyoverdine in *P. aeruginosa*, and strains without PA2412 cannot produce pyoverdine or grow under iron-restricted conditions (Drake et al., 2007). Furthermore, PA2412 has the ability to promote ENT biosynthesis in *E. coli* in the absence of YbdZ (Schomer and Thomas, 2017). Intriguingly, the orphan MLP MXAN_3118 from *M. xanthus* is not encoded within any NRP BGC, but it is able to interact with seven different NRPSs that are encoded elsewhere in the genome of this organism (Esquilín-Lebrón et al., 2018). In addition, MXAN_3118 can functionally replace YbdZ in multiple assays conducted in *E. coli* (Schomer and Thomas, 2017) and is therefore thought to be a promising “universal” MLP for promoting heterologous expression of NRPSs in bacterial and fungal strains in order to improve metabolite production.

In addition to MLPs with known function, we chose MLP candidates for our study that have not been previously characterized and which are associated with predicted NRP BCGs (Supplementary Figure 3). Three (CGL27_RS10110, CGL27_RS02360, and AWZ11_RS05060) are from the plant pathogenic species *S. europaeiscabiei* (Zhang et al., 2016) and *Streptomyces* sp. 11-1-2 (Bown and Bignell, 2017), and one (RHA1_ro04717) is from the actinobacterium *R. jostii*, which is known for its ability to transform a variety of organic compounds and pollutants (Martínková et al., 2009). In addition, we included SCLAV_p1293, which is associated with a predicted BGC on the linear plasmid of *S. clavuligerus* and was previously found to be unable to promote thaxtomin production in the *S. scabiei* MLP triple mutant (Li et al., 2019a).

### Non-cognate MLPs From Different Bacteria Can Promote the Solubility of the TxtA and TxtB A-Domains to Varying Degrees

Previously, we showed that TxtH is required for the soluble production of both TxtA^*A*^ and TxtB^*A*^ in *E. coli*, suggesting that it functions as a chaperone to promote the proper folding of the NRPS adenylating enzymes. Two non-cognate MLPs encoded elsewhere on the *S. scabiei* chromosome were also shown to be able to promote the soluble production of TxtA^*A*^ and TxtB^*A*^, suggesting that some MLPs can exhibit functional redundancy with TxtH (Li et al., 2019a). To determine whether non-cognate MLPs from other bacterial species are able to exhibit functional cross-talk with TxtH, we expressed each A-domain with an N-terminal HIS_6_ tag together or without an MLP, which also harbored an N-terminal HIS_6_ tag. The amount of HIS_6_-tagged TxtA^*A*^ and TxtB^*A*^ when co-expressed with each MLP was then assessed in soluble protein fractions by western blot analysis using antibodies against the HIS_6_ tag.

**FIGURE 2:**
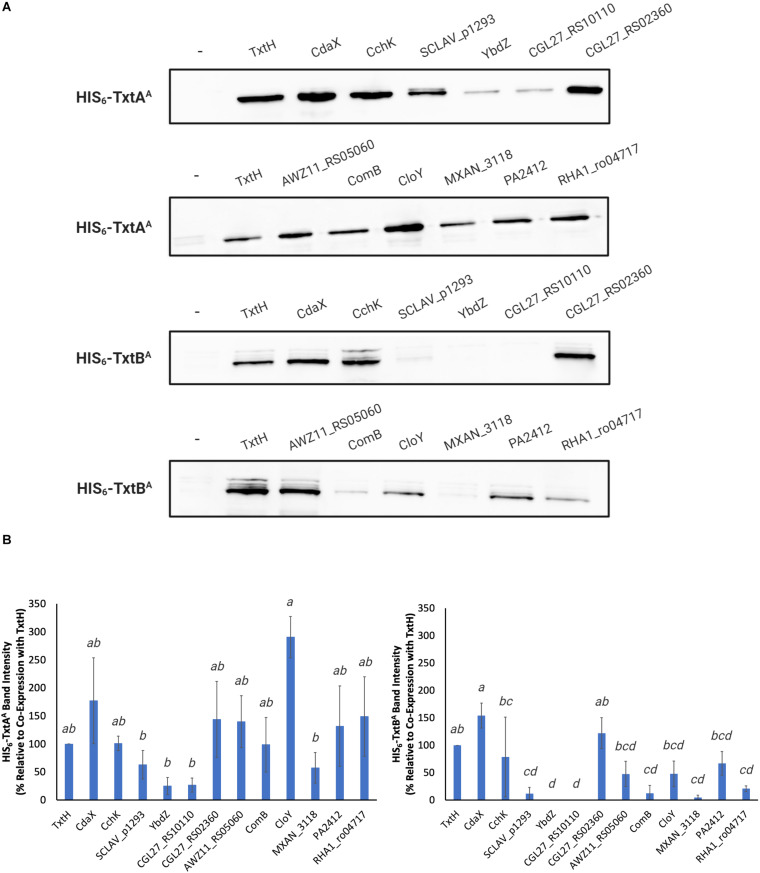
**(A)** Western blot analysis of soluble HIS_6_-TxtA^*A*^ and HIS_6_-TxtB^*A*^ proteins expressed in the presence and absence (–) of different HIS_6_-tagged MLPs in *E. coli* BL21(DE3)*ybdZ:aac(3)IV*. The analysis was conducted three times, and one representative set of blots is shown. **(B)** Quantification of the HIS_6_-TxtA^*A*^ (left) and HIS_6_-TxtB^*A*^ (right) protein band intensities following co-expression with different HIS_6_-tagged MLPs. The bars represent the mean percent band intensity from triplicate western blots relative to the control (co-expression with HIS_6_-TxtH; set to 100%) and was determined using ImageJ. Error bars represent the standard deviation from the mean. Means with different letters (*a*–*d*) were determined to be significantly different (*P* ≤ 0.05).

Compared to TxtH, the non-cognate MLPs promoted the production of the two A-domains in soluble form with varying efficiencies (Figure 2A). In the case of TxtA^*A*^, co-expression with SCLAV_p1293, YbdZ, CGL27_RS10110, and MXAN_3118 resulted in reduced soluble protein production, though the observed differences were not statistically significant when compared with the TxtH co-expression (Figure 2B). In contrast, the remaining MLPs promoted similar or higher soluble TxtA^*A*^ protein levels (Figures 2A,B). For TxtB^*A*^, co-expression with CdaX, CchK, and CGL27_RS02360 resulted in similar or higher amounts of soluble protein production when compared to the TxtH co-expression. However, the remaining MLPs failed, or promoted reduced levels of soluble TxtB^*A*^ production, with most resulting in statistically significant differences in protein levels when compared to TxtH (Figures 2A,B). Production of both A-domains in soluble form was most severely impacted by co-expression with YbdZ and CGL27_RS10110, followed by SCLAV_p1293 and MXAN_3118. Of the two Txt NRPS A-domains, the soluble production of TxtB^*A*^ was more strongly impacted by the different co-expressed MLP partners (Figures 2A,B). This is in accordance with previous reports showing differences in MLP-NRPS A-domain interactions, even within the same NRP biosynthetic pathway involving multiple NRPS enzymes (Felnagle et al., 2010; McMahon et al., 2012; Davidsen et al., 2013). Although there was some variability in the relative expression level of the MLPs in the *E. coli* strain based on SDS-PAGE analysis of the total soluble protein extracts (Supplementary Figure 1), we found no correlation between the amount of MLP detected and the amount of soluble A-domain protein produced when co-expressed with the MLP. For example, SCLAV_p1293 and MXAN_3118 were both detected at higher levels than TxtH in the total protein extracts, but neither were able to promote efficient production of soluble TxtB^*A*^. In contrast, CGL27_RS02360 was not readily detectable in the total extracts, but it was able to promote the soluble production of both A-domains to levels comparable to those observed with TxtH. Our observations are consistent with other studies that also found no correlation between the detectable level of an MLP and its ability to promote soluble A-domain protein production in *E. coli* (Schomer and Thomas, 2017; Schomer et al., 2018). Overall, our results show that several phylogenetically distinct MLPs have the ability to functionally replace TxtH in promoting the soluble production of the Txt NRPS adenylating enzymes in *E. coli* to varying degrees, though not all MLPs are able to do so.

### Influence of Non-cognate MLPs on Thaxtomin Production in *S. scabiei*

**FIGURE 3:**
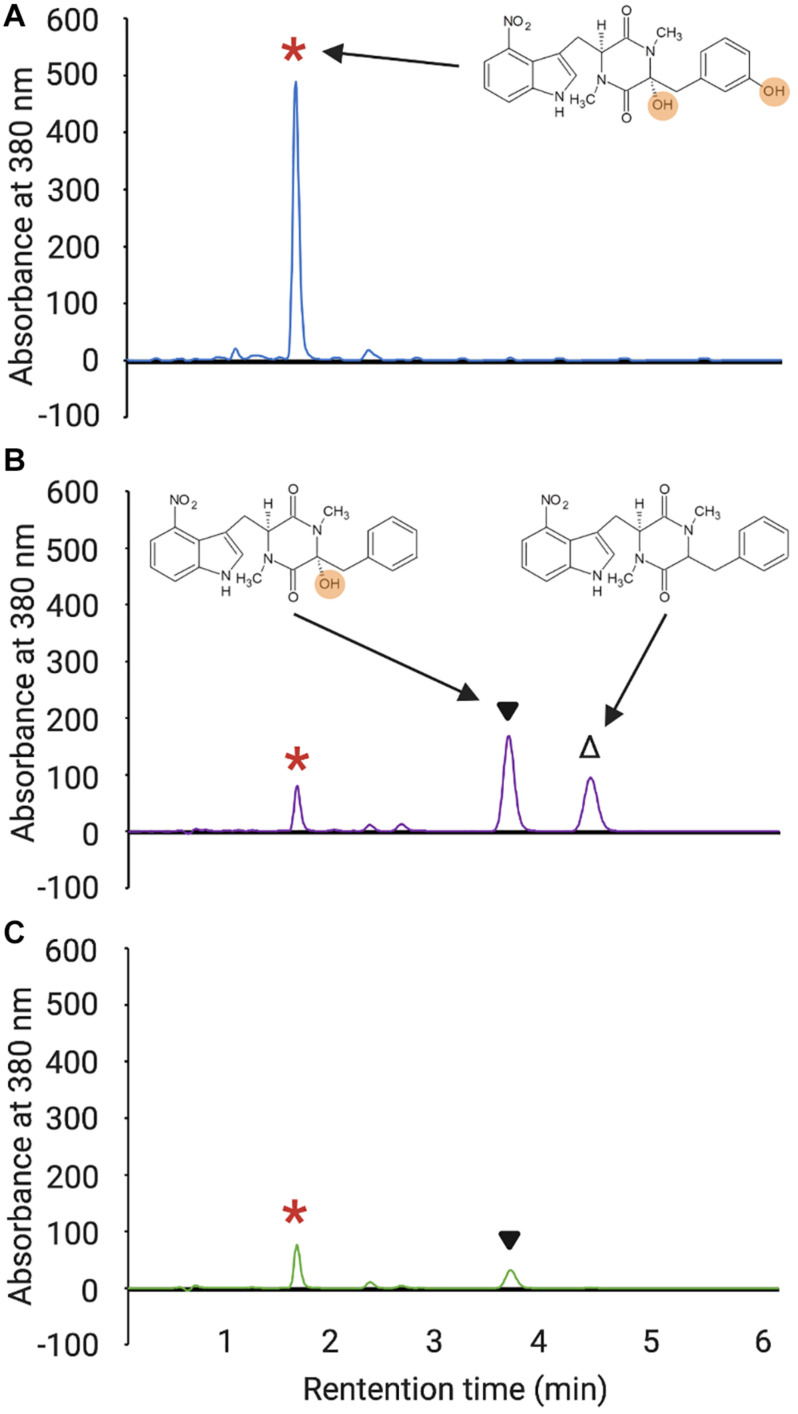
HPLC analysis of culture extracts from wild-type *S. scabiei* 87.22 **(A)**, the *S. scabiei* triple MLP deletion mutant (Δ*mlp*_*lipo*_/Δ*txtH*/Δ*mlp*_*scab*_) containing the *txtH* expression vector **(B)** and the triple MLP deletion mutant containing the *MXAN_3118* expression vector **(C)**. The peak corresponding to thaxtomin A (retention time = 1.65 min) in each chromatogram is indicated with the red asterisks, and the peaks corresponding to thaxtomin B (retention time = 3.81 min) and thaxtomin D (retention time = 4.61 min) are indicated with ▼and Δ, respectively. The chemical structures of thaxtomin A, B, and D are also shown next to the corresponding peaks, and the hydroxyl groups of thaxtomin A and B are highlighted.

In addition to examining the impact of the non-cognate MLPs on Txt NRPS A-domain solubility, we assessed their ability to promote the production of thaxtomin A in the absence of the native MLPs in *S. scabiei.* This was accomplished by overexpressing each MLP in a *S. scabiei* mutant that lacks all three endogenous MLP-encoding genes, including *txtH*, and is unable to produce thaxtomin (Li et al., 2019a). As reported previously, overexpression of *txtH* restores thaxtomin A production in the mutant, though not to levels observed in the wild-type strain (Figures 3A,B). This is due to polar effects of the *txtH* mutation on expression of the downstream *txtC* gene (Li et al., 2019a), which encodes the P450 monooxygenase that hydroxylates the thaxtomin backbone at the α- and/or ring carbon of the phenylalanine moiety (Healy et al., 2002; Alkhalaf et al., 2019). In addition, the thaxtomin B and D intermediates, which differ from thaxtomin A in the absence of one or both of the TxtC-dependent hydroxyl groups, were found to accumulate in the *S. scabiei* MLP triple mutant when *txtH* was overexpressed (Figure 3B), which is consistent with the observed polar effects of the *txtH* mutation on *txtC* gene expression. Therefore, in order to evaluate the efficiency of the different MLPs to exhibit functional redundancy with TxtH, the combined production of thaxtomins (thaxtomin A, B, D) was assessed in each of the MLP overexpression strains to account for any polar effects.

**FIGURE 4:**
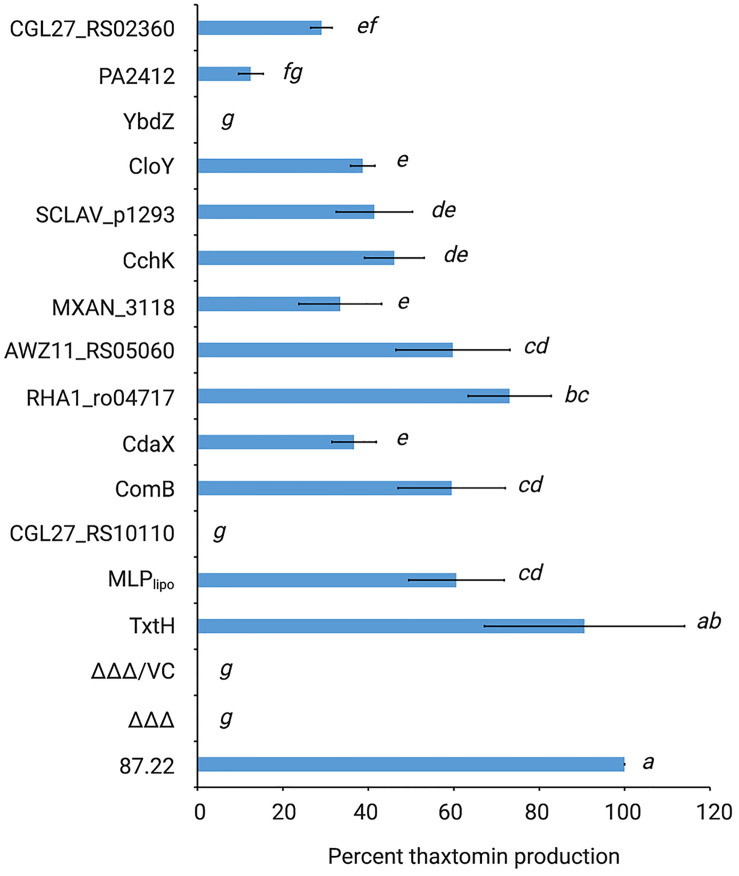
Relative quantification of thaxtomin production in the *S. scabiei* MLP triple mutant (Δ*mlp*_*lipo*_/Δ*txtH*/Δ*mlp*_*scab*_; ΔΔΔ) expressing different non-cognate MLPs. The production levels are represented as the average% production of thaxtomins (thaxtomin A, thaxtomin B, and thaxtomin D) relative to wild-type *S. scabiei* 87.22 (±SD). *n* = 3 biological replicates for 87.22, ΔΔΔ, ΔΔΔ/VC (vector control); *n* = 5 biological replicates for SCLAV_p1293; and *n* = 6 biological replicates for all other strains. Means with different letters (*a*–*g*) were determined to be significantly different (*P* ≤ 0.05).

As shown in Figure 4, all but two of the non-cognate MLPs were able to restore thaxtomin production in the MLP triple mutant to varying degrees. Overexpression of RHA1_ro04717 was most effective at restoring production to levels similar to that observed for TxtH, while overexpression of AWZ11_RS05060 and ComB restored production to levels similar to that observed for MLP_*lipo*,_ a non-cognate MLP in *S. scabiei* that was previously shown to exhibit functional cross-talk with TxtH (Li et al., 2019a). The overexpression of CloY, MXAN_3118, CdaX, CchK, SCLAV_p1293, CGL27_RS02360, and PA2412 led to partial complementation of thaxtomin production, with levels ranging from 14–51% of that observed for TxtH (Figure 4). Among the MLPs tested, only YbdZ and CGL27_RS10110 were unable to restore detectable thaxtomin production when overexpressed in the MLP triple mutant. Interestingly, all three thaxtomins (thaxtomin A, B, D) were present in culture extracts of successfully complemented MLP strains with the exception of the MXAN_3118 overexpression strain, which did not accumulate detectable levels of thaxtomin D (Figure 3C). The reason for this is currently unclear, but it warrants further investigation.

It is noteworthy that the results observed for CdaX and SCLAV_p1293 are contradictory to the results of our previous study, which found that overexpression of both genes failed to complement thaxtomin production in the *S. scabiei* MLP triple mutant (Li et al., 2019a). The reason behind this discrepancy is not clear, but it could be due to differences in the *Streptomyces* expression vectors that were used. In the current study, we used pRFSRL16, which harbors the *ermE*p^∗^ promoter as well as a Shine-Dalgarno (SD) sequence (AAAGGAGG) for expression of the cloned gene. In contrast, the expression vector used in our previous study (pRLDB50-1a) contains the *ermE*p^∗^ promoter but no SD sequence, and thus the native SD sequence was cloned along with the coding sequence of the gene to be expressed. As translation initiation is considered the rate limiting step of protein synthesis in bacteria, and there is evidence that the SD sequence and context play an important role in the initiation of translation of many mRNA transcripts (Gualerzi and Pon, 2015), it is possible that the different expression vectors used in the current and previous study contributed to differences in levels of the CdaX and SCLAV_p1293 proteins produced in *S. scabiei*, though further investigations are required to verify this.

**FIGURE 5:**
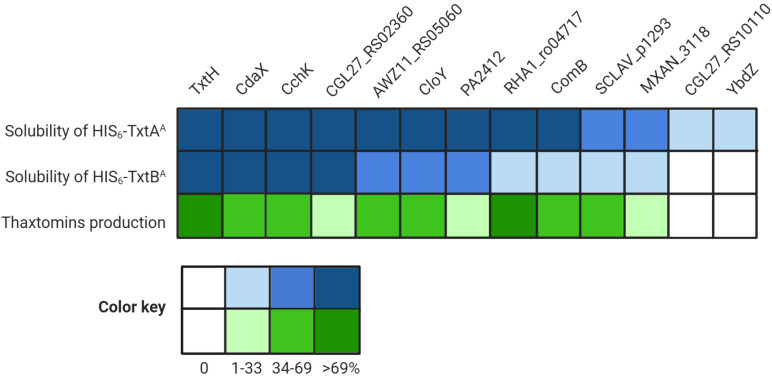
Summary of the results of the different assays examining the interaction between the thaxtomin NRPSs and the non-cognate MLPs. The heat map illustrates the relative amount of soluble HIS_6_-TxtA^*A*^ and HIS_6_-TxtB^*A*^ proteins produced in the presence of the different MLPs as compared to TxtH (set to 100%), as well as the relative thaxtomin production levels in the presence of different MLPs as compared to TxtH (set to 100%).

The results of the thaxtomin analysis together with the protein solubility assay are summarized in Figure 5. In general, the ability of an MLP to promote the soluble production of the Txt NRPS A-domains in *E. coli* corresponded with its ability to promote thaxtomin production in *S. scabiei.* In other words, only MLPs that enabled the soluble production of both A-domains, even in low amounts, were also found to promote the detectable production of thaxtomins. The ability of an MLP to serve as a functional partner was not correlated with amino acid similarity, since the two MLPs (YbdZ and CGL27_RS10110) that were unable to exhibit functional cross-talk with TxtH were just as similar to TxtH as MLPs that could exhibit functional cross-talk (Table 3). A similar phenomenon was reported by Schomer and Thomas (2017), who found that the ability of non-cognate MLPs to compensate for the loss of YbdZ in *E. coli* did not correlate with the similarity of the MLP to YbdZ.

It is notable that the relative efficiency of soluble protein production by an MLP did not correlate well with the relative efficiency of thaxtomin production in our study. For example, CdaX, CchK, and CGL27_RS02360 were all able to promote the production of soluble protein for both of the Txt NRPS A-domains at levels similar to or great than that observed in the presence of TxtH, and yet none were able to fully complement thaxtomin production in the *S. scabiei* MLP triple mutant. Similarly, PA2412, CloY and AWZ11_RS05060 exhibited somewhat comparable protein solubility profiles for both A-domains, but PA2412 was significantly less efficient at promoting thaxtomin production. PA2412 was also less efficient at promoting thaxtomin production than MXAN_3118, but it was more efficient at promoting the soluble production of both of the Txt A-domains than MXAN_3118. In addition, RHA1_ro04717 was the only non-cognate MLP that was able to fully complement thaxtomin production in *S. scabiei*, but it was much less efficient at promoting the soluble production of TxtB^*A*^ compared to some other MLPs. While it is plausible that the solubility-promoting activity of some MLPs in our co-expression assay may have been influenced by the presence of the N-terminal HIS_6_ tag, we previously showed that the HIS_6_ tag does not impact this activity in the case of TxtH (Li et al., 2019a). Overall, our results suggest that the efficiency at which an MLP is able to promote NRPS A-domain solubility is not always a reliable indicator of the relative functionality of the MLP-NRPS pair *in vivo.* This may be due to effects of the MLP on the folding of the entire NRPS machinery that are not revealed when examining the individual A-domains alone. In addition, other studies have found that MLPs have a broader impact on NRPSs beyond protein solubility (Heemstra et al., 2009; Felnagle et al., 2010; Zhang et al., 2010; Boll et al., 2011; Miller et al., 2016; Schomer and Thomas, 2017; Schomer et al., 2018; Mori et al., 2018a). Schomer and Thomas (2017) showed that non-cognate MLPs can influence the solubility and catalysis of the EntF NRPS, including aminoacyl-*S-*PCP formation, and that these effects are separable. PA2412, for example, can enhance the catalysis of EntF but has no impact on EntF solubility, whereas two other non-cognate MLPs (CmnN, VioN) can enhance EntF solubility but do not influence catalysis. To date, we have been unable to detect the production of soluble Txt A-domain protein in the absence of TxtH, and so the effect of TxtH or other MLPs on the adenylation or other activities of the TxtA and/or TxtB NRPS enzymes is currently unknown.

### *In silico* Analysis of the MLP-NRPS Interface Involved in Thaxtomin Biosynthesis

**FIGURE 6:**
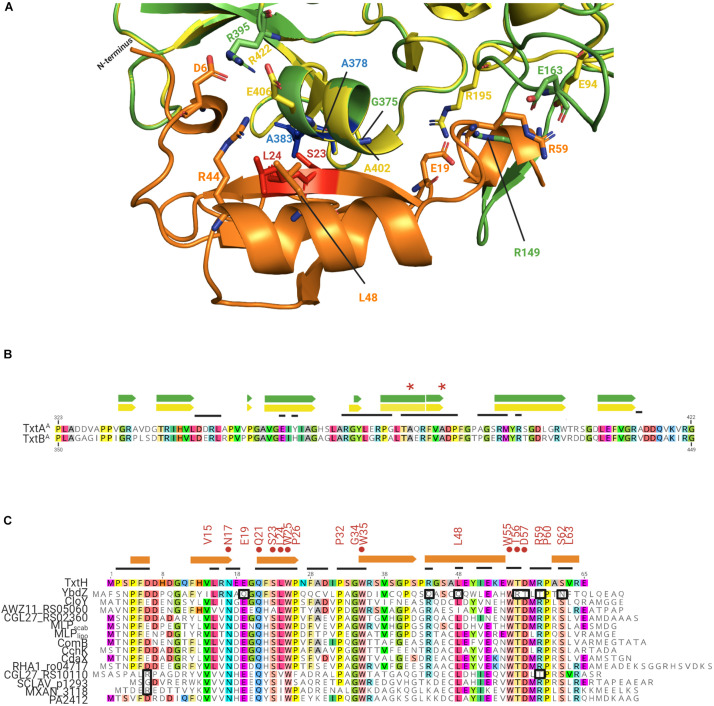
**(A)** Predicted interaction interface between the *S. scabiei* Txt A-domains and TxtH. TxtA^*A*^ is shown in green, TxtB^*A*^ is shown in yellow, and TxtH is shown in orange. The strictly conserved serine and leucine residues (red) of TxtH (S23 and L24) and two possible interacting alanine residues (blue) of TxtA^*A*^ (A378 and A383) are highlighted. The corresponding alanine residues of TxtB^*A*^ (A405 and A410) are not labeled. The residues that are associated with interaction interface are shown as sticks. **(B)** Partial amino acid sequence alignment of the *S. scabiei* TxtA^*A*^ and TxtB^*A*^. The residues involved in the formation of α-helix (box) and β-sheets (arrows) within the predicted structures are indicated above the alignment in green (for TxtA^*A*^) and yellow (for TxtB^*A*^). Residues in TxtA^*A*^ and TxtB^*A*^ that fall within the interaction interface between the TioS/FscH NRPSs and their cognate MLPs (TioT/FscK) are indicated by the black lines above the amino acid alignment. The two alanine residues of TxtA^*A*^ and TxtB^*A*^ that are predicted to interact with S23 and L24 of TxtH are indicated by the astericks. **(C)** Amino acid sequence alignment of TxtH from *S. scabiei* and the non-cognate MLPs from other bacteria that were analyzed in this study. Residues within the non-cognate MLPs that match the amino acid residue in TxtH at the same position are colored. The consensus sequence (VxxNxExQxSLWP-x5-PxGW-x12-L-x6-WTDxRPxSL) appearing in more than 85% of the 133 MLPs used in the phylogenetic analysis are indicated above the alignment. The residues shown to be important for the soluble production of TxtA^*A*^ and/or TxtB^*A*^ by TxtH (Li et al., 2019a) are indicated by the red circles. Variant residues in the non-cognate MLPs that may have a negative impact on the interaction with the thaxtomin NRPSs are highlighted in black boxes. Extracted secondary structures for TxtH are shown using orange boxes (helixes) and arrows (β-sheets) above the alignment. Residues in TxtH that fall within the interaction interface between TioT/FscK and their corresponding NRPSs (TioS/FscH) are indicated by the black lines above the amino acid alignment.

Although the degree of amino acid similarity between non-cognate MLPs and TxtH is unable to fully explain why some MLPs are capable of exhibiting functional cross-talk with TxtH while others are not, the overall topology of the Txt MLP-NRPS protein complex interface could provide some insights. Therefore, we utilized SWISS-MODEL to create *in silico* models for TxtA^*A*^, TxtB^*A*^, and TxtH using the structures of protein templates (Supplementary Table 3) that exhibited the best scores for GMQE (Global Model Quality Estimation) and QMEAN (Qualitative Model Energy Analysis; Benkert et al., 2011; Waterhouse et al., 2018). Specifically, the structural models of TxtA^*A*^ and TxtB^*A*^ (Supplementary Figure 4) were computationally generated using the solved structure of the TioS NRPS from the thiocoraline biosynthetic pathway of *Micromonospora* sp. ML1 (PDB ID: 5wmm_1) as the template. The TioS NRPS requires its cognate MLP TioT for soluble production in *E. coli*, and the structure of the protein complex (PDB ID: 5wmm) revealed that TioT interacts with helix 10 and beta strands 18 and 19 from the A-domain of TioS (Mori et al., 2018b). TxtH was modeled using the crystal structure of the FscK MLP from *T. fusca* (PDB ID: 6ea3_1) as the template. The predicted TxtH structure is composed of three stranded anti-parallel beta sheets, one alpha helix and two single turn helices at its two termini (Supplementary Figure 4), which resembles the typical MLP monomers of solved structures (Drake et al., 2007; Miller et al., 2016; Tarry et al., 2017). During the modeling analysis, TxtH was docked with TxtA^*A*^ or TxtB^*A*^ based on the location of TioT in the TioT-TioS complex (Figure 6A and Supplementary Figure 4). The predicted TxtH-TxtA^*A*^/B^*A*^ interface is highly similar to that seen with other reported MLP-NRPS complexes (Herbst et al., 2013; Miller et al., 2016; Tarry et al., 2017; Mori et al., 2018b), where residues S23 and L24 of TxtH are predicted to hydrogen bond with A383 and A378 of TxtA^*A*^, and with A410 and A405 of TxtB^*A*^ (Figure 6A). The same interaction is also observed in the adenylating enzyme SlgN1 from *Streptomyces lydicus*, which contains an MLP domain at its N-terminus (Herbst et al., 2013). Notably, the importance of residues S23 and L24 for the solubility-promoting activity of TxtH has been substantiated by site-directed mutagenesis (Li et al., 2019a).

The predicted TxtA^*A*^ and TxtB^*A*^ structures display some differences, however, both models can make contacts with TxtH (Figure 6A and Supplementary Figure 4). The predicted TxtH binding interface region involves residues from helix 16 and beta sheets 19-22 of TxtA^*A*^, and helix 15 and beta sheets 17–20 of TxtB^*A*^ (Figures 6A,B). Several variable residues are present within the interface region of TxtA^*A*^ and TxtB^*A*^ (Figure 6B), suggesting that the two Txt NRPSs may interact differently with MLP partners, including TxtH. This is in line with results from the current study, where the solubility of TxtB^*A*^ was impacted more than that of TxtA^*A*^ by the MLP partner that it was co-expressed with (Figures 2A,B). In addition, our previous work showed that the solubility of TxtB^*A*^ was affected to a greater extent than TxtA^*A*^ during co-expression with various TxtH point mutants (Li et al., 2019a). Therefore, our results suggest that the formation of an MLP-NRPS functioning pair involves a more stringent interaction in the case of TxtB than it does for TxtA.

More detailed analysis of the amino acid sequences of the 133 MLP proteins used in the phylogenetic analysis (Figure 1) indicated the presence of a sequence/motif (VxxNxExQxSLWP-x5-PxGW-x12-L-x6-WTDxRPxSL) in >85% of the proteins. The motif is similar to the signature sequence that was previously proposed by Baltz (2011) for predicting functional MLP homologues in sequenced genomes. FscK, the MLP whose structure (PDB ID: 6ea3_1) was used as a template to model TxtH, also contains all the residues from the motif. Many of these residues (except for V15, E19 and D57) are situated at its NRPS interacting interface, suggesting their importance for MLP functionality. In addition, the motif is well conserved in TxtH (except for L63) and in some of the non-cognate MLPs examined in the current study (Figure 6C), whereas other proteins display some variations. It is possible that differences in the sequence of this motif along with differences at other positions might impact the interaction of MLPs with one or both of the Txt A-domains. For instance, the positively-charged R44 residue of TxtH is predicted to form a salt bridge with E406 in TxtB^*A*^ based on homology modeling using the MLP-NRPS structures of TioT-TioS or SlgN1 as template (Herbst et al., 2013; Mori et al., 2018b). In the case of YbdZ, the corresponding residue is an uncharged Q (Figure 6C), which is not expected to be involved in salt bridge formation and could potentially impact the YbdZ-TxtB^*A*^ interaction. Therefore, the R→Q substitution in YbdZ might explain why this non-cognate MLP failed to promote the soluble expression of TxtB^*A*^ (Figure 2). On the other hand, SCLAV_p1293, MXAN_3118, and PA2412 contain a positively charged K residue at the same position (Figure 6C) and promoted soluble TxtB^*A*^ protein production, but not to the same extent as TxtH (Figure 2). It has been reported that RE salt bridges are more favorable for speeding up protein folding as compared to KE (Meuzelaar et al., 2016), but their relevance in the MLP-TxtA/B interaction requires further investigation.

Another potential interaction could involve the negatively-charged E19 residue of TxtH, which is predicted to be in close proximity to R149 in TxtA^*A*^ and R195 in TxtB^*A*^ (Figure 6A). The TxtH E19 residue is conserved in all of the non-cognate MLPs with the exception of YbdZ, which contains an uncharged Q at that position (Figure 6C), and could be another reason for the inability of YbdZ to promote Txt NRPS A-domain solubility (Figures 2A,B). In TxtH, D6 is predicted to contribute to hydrogen bonding and salt bridge formation with R395 in TxtA^*A*^ and R422 in TxtB^*A*^ to stabilize the MLP-NRPS interface (Figure 6A). A similar interaction is observed between D7 of TioT and R395 of TioS, between E6 of the MLP domain and R446 of the A-domain in SlgN1, as well as between D1324 of the MLP domain and R853 of the A-domain in ObiF1 (Herbst et al., 2013; Mori et al., 2018b; Kreitler et al., 2019). It should be noted that the corresponding negatively charged D or E residues are not present in either CGL27_RS10110, SCLAV_p1293, and MXAN_3118, which could in part explain why some of them failed or were not as efficient as TxtH in promoting Txt NRPS A-domain solubility or thaxtomin production (Figures 2, 4).

In general, the C-terminal region of the conserved motif in YbdZ (Q-x6-WRTxTPxN) differs significantly from that present in TxtH (L-x6-WTDxRPxS) and other non-cognate MLPs (Figure 6C). In FscK and TioT, residues from this region (with the exception of D) are involved in binding with their cognate NRPS partners (Bruner and Zagulyaeva, unpublished; Mori et al., 2018b). In our model, the hydrophobic side chain of L48 from TxtH is closely packed with G375 in TxtA^*A*^ and A402 in TxtB^*A*^ toward the center of the interface, possibly contributing to non-polar interactions (Figure 6A). The substitution of a polar Q residue at this position in YbdZ may further hinder its interaction with the Txt A-domain proteins from the current study (Figure 6C). In addition, the solution structure of the Rv2377c MLP from *M. tuberculosis* and of PA2412 from *P. aeruginosa* has demonstrated that the highly conserved WTDxRP portion of the motif is within an intrinsically disordered region in both proteins (Buchko et al., 2010). Disordered regions of proteins have been associated with functional diversity or with binding to multiple protein partners (Haynes et al., 2006; Xie et al., 2007). In our previous work, we showed that the WTD residues of TxtH are all important for promoting the solubility of TxtA^*A*^ and TxtB^*A*^ (Li et al., 2019a). The WTDxRP motif is absolutely conserved in all of the non-cognate MLPs examined in our studies except for YbdZ and CGL27_RS10110, both of which contain a T instead of the R residue (Figure 6C). R59 of TxtH is predicted to form a salt bridge with E163 of TxtA^*A*^ and E94 of TxtB^*A*^ (Figure 6A), and the substitution to an uncharged T may impact the ability of YbdZ and CGL27_RS10110 to bind efficiently to the A-domains, which could further explain they were not able to replace TxtH in the assays conducted (Figure 5). Overall, the structures of MLPs and their partners (including our *in silico* TxtH-TxtA^*A*^/B^*A*^ models) provide important insights into the key residues that are involved in MLP/NRPS interactions and which may also account for the ability of MLPs from different biosynthetic pathways to exhibit functional redundancy. The question of why functional cross-talk occurs among different MLPs and its significance is one that remains to be addressed.

## Concluding Remarks

Here, we showed that phylogenetically distinct MLPs from different organisms vary in their ability to exhibit functional redundancy with TxtH from the thaxtomin biosynthetic pathway in *S. scabiei.* Except for YbdZ and CGL27_RS10110, all MLPs examined in this study were able to promote the soluble production of the Txt A-domains in *E. coli* and enabled thaxtomin production to varying degrees in a *S. scabiei* mutant lacking endogenous MLPs. *In silico* structural analysis of TxtH with its cognate NRPS A-domains revealed that the ability of different non-cognate MLPs to exhibit functional cross-talk with TxtH likely depends on the conservation of key residues at the MLP-NRPS interaction interface rather than the overall amino acid similarity shared between the proteins. In addition, the *in silico* analysis combined with our protein solubility assay results suggest that the two Txt NRPSs differ in their interactions with TxtH and with most of the non-cognate MLPs examined in this study. Overall, our study provides additional insights into the mechanism of MLP cross-talk and its impact on specialized metabolite biosynthesis in bacteria. Thaxtomin A is essential for common scab disease development by *S. scabiei* and other plant pathogenic *Streptomyces* spp., and thus our research on the thaxtomin biosynthetic machinery is expected to have useful applications for the development of strategies for effective disease management. Furthermore, the potent herbicidal activity exhibited by thaxtomin A (King et al., 2001) makes it an attractive bioherbicide for controlling the growth of weeds (Leep et al., 2010; Koivunen and Marrone, 2013), and a better understanding of the thaxtomin biosynthetic pathway may facilitate the large-scale commercial production of this compound for agricultural applications. Currently, work is ongoing to determine whether TxtH and the non-cognate MLPs examined in this study can influence the catalytic activity of either or both of the Txt NRPSs. In addition, the crystal structure of the TxtH-TxtA(B) complexes will be useful in better understanding the molecular basis for the interaction between TxtH and its two cognate NRPSs. Finally, the ability of TxtH and other non-cognate MLPs to influence the production of other NRPs in *S. scabiei* is the subject of on-going studies.

## Data Availability Statement

The raw data supporting the conclusions of this article will be made available by the authors, without undue reservation.

## Author Contributions

The study concept and experimental methodology were designed by YL, DB, and KT. All experimental procedures and data analyses were conducted by YL, and the manuscript was written by YL with editorial input from DB and KT. All authors contributed to the article and approved the submitted version.

## Conflict of Interest

The authors declare that the research was conducted in the absence of any commercial or financial relationships that could be construed as a potential conflict of interest.

## Footnotes

1 https://www.idtdna.com/codonopt

2 http://blast.ncbi.nlm.nih.gov/Blast.cgi

3 https://itol.embl.de/

4 https://swissmodel.expasy.org/

5 https://www.ebi.ac.uk/pdbe/pisa/

## References

[B1] Alkhalaf L. M. Barry S. M. Rea D. Gallo A. Griffiths D. Lewandowski J. R. (2019). Binding of distinct substrate conformations enables hydroxylation of remote sites in thaxtomin D by cytochrome P450 TxtC. *J. Am. Chem. Soc.* 141 216–222. 10.1021/jacs.8b08864 30516965

[B2] Al-Mestarihi A. H. Villamizar G. Fernaìndez J. Zolova O. E. Lomboì F. Garneau-Tsodikova S. (2014). Adenylation and S-methylation of cysteine by the bifunctional enzyme TioN in thiocoraline biosynthesis. *J. Am. Chem. Soc.* 136 17350–17354. 10.1021/ja510489j 25409494

[B3] Baltz R. H. (2011). Function of MbtH homologs in nonribosomal peptide biosynthesis and applications in secondary metabolite discovery. *J. Ind. Microbiol. Biotechnol.* 38:1747. 10.1007/s10295-011-1022-8 21826462

[B4] Benkert P. Biasini M. Schwede T. (2011). Toward the estimation of the absolute quality of individual protein structure models. *Bioinformatics* 27 343–350. 10.1093/bioinformatics/btq662 21134891 PMC3031035

[B5] Biasini M. Bienert S. Waterhouse A. Arnold K. Studer G. Schmidt T. (2014). SWISS-MODEL: modelling protein tertiary and quaternary structure using evolutionary information. *Nucleic Acids Res.* 42 W252–W258. 10.1093/nar/gku340 24782522 PMC4086089

[B6] Boll B. Taubitz T. Heide L. (2011). Role of MbtH-like proteins in the adenylation of tyrosine during aminocoumarin and vancomycin biosynthesis. *J. Biol. Chem.* 286 36281–36290. 10.1074/jbc.M111.288092 21890635 PMC3196098

[B7] Bown L. Bignell D. R. D. (2017). Draft genome sequence of the plant pathogen *Streptomyces* sp. *strain* 11-1-2. *Genome Announc.* 5:e00968-17. 10.1128/genomeA.00968-17 28912321 PMC5597762

[B8] Buchko G. W. Kim C. Y. Terwilliger T. C. Myler P. J. (2010). Solution structure of Rv2377c-founding member of the MbtH-like protein family. *Tuberculosis* 90 245–251. 10.1016/j.tube.2010.04.002 20434955 PMC2910232

[B9] Chiu H. T. Hubbard B. K. Shah A. N. Eide J. Fredenburg R. A. Walsh C. T. (2001). Molecular cloning and sequence analysis of the complestatin biosynthetic gene cluster. *Proc. Natl. Acad. Sci. U.S.A.* 98 8548–8553. 10.1073/pnas.151246498 11447274 PMC37473

[B10] Davidsen J. M. Bartley D. M. Townsend C. A. (2013). Non-ribosomal propeptide precursor in nocardicin A biosynthesis predicted from adenylation domain specificity dependent on the MbtH family protein NocI. *J. Am. Chem. Soc.* 135 1749–1759. 10.1021/ja307710d 23330869 PMC3571714

[B11] DeLano W. L. (2002). Pymol: an open-source molecular graphics tool. *CCP4 Newsl. Protein Crystallogr.* 40 82–92.

[B12] Drake E. J. Cao J. Qu J. Shah M. B. Straubinger R. M. Gulick A. M. (2007). The 1.8 Å crystal structure of PA2412, an MbtH-like protein from the pyoverdine cluster of *Pseudomonas aeruginosa*. *J. Biol. Chem.* 282 20425–20434. 10.1074/jbc.M611833200 17502378

[B13] Esquilín-Lebrón K. J. Boynton T. O. Shimkets L. J. Thomas M. G. (2018). An orphan MbtH-Like protein interacts with multiple nonribosomal peptide synthetases in *Myxococcus xanthus* DK1622. *J. Bacteriol.* 200:e00346-18. 10.1128/JB.00346-18 30126939 PMC6182236

[B14] Felnagle E. A. Barkei J. J. Park H. Podevels A. M. McMahon M. D. Drott D. W. (2010). MbtH-like proteins as integral components of bacterial nonribosomal peptide synthetases. *Biochemistry* 49 8815–8817. 10.1021/bi1012854 20845982 PMC2974439

[B15] Finking R. Marahiel M. A. (2004). Biosynthesis of nonribosomal peptides. *Annu. Rev. Microbiol.* 58 453–488. 10.1146/annurev.micro.58.030603.123615 15487945

[B16] Fyans J. K. Bown L. Bignell D. R. D. (2016). Isolation and characterization of plant-pathogenic *Streptomyces* species associated with common scab-infected potato tubers in Newfoundland. *Phytopathology* 106 123–131. 10.1094/PHYTO-05-15-0125-R 26524546

[B17] Gualerzi C. O. Pon C. L. (2015). Initiation of mRNA translation in bacteria: structural and dynamic aspects. *Cell. Mol. Life. Sci.* 72 4341–4367. 10.1007/s00018-015-2010-3 26259514 PMC4611024

[B18] Haynes C. Oldfield C. J. Ji F. Klitgord N. Cusick M. E. Radivojac P. (2006). Intrinsic disorder is a common feature of hub proteins from four eukaryotic interactomes. *PLoS Comput. Biol.* 2:e100. 10.1371/journal.pcbi.0020100 16884331 PMC1526461

[B19] Healy F. G. Krasnoff S. B. Wach M. Gibson D. M. Loria R. (2002). Involvement of a cytochrome P450 monooxygenase in thaxtomin A biosynthesis by *Streptomyces acidiscabies*. *J. Bacteriol.* 184 2019–2029. 10.1128/JB.184.7.2019-2029.2002 11889110 PMC134914

[B20] Heemstra J. R. Walsh C. T. Sattely E. S. (2009). Enzymatic tailoring of ornithine in the biosynthesis of the *Rhizobium* cyclic trihydroxamate siderophore vicibactin. *J. Am. Chem. Soc.* 131 15317–15329. 10.1021/ja9056008 19778043 PMC2783850

[B21] Herbst D. A. Boll B. Zocher G. Stehle T. Heide L. (2013). Structural basis of the interaction of MbtH-like proteins, putative regulators of nonribosomal peptide biosynthesis, with adenylating enzymes. *J. Biol. Chem.* 288 1991–2003. 10.1074/jbc.M112.420182 23192349 PMC3548506

[B22] Huguet-Tapia J. C. Lefebure T. Badger J. H. Guan D. Pettis G. S. Stanhope M. J. (2016). Genome content and phylogenomics reveal both ancestral and lateral evolutionary pathways in plant-pathogenic *Streptomyces* species. *Appl. Environ. Microbiol.* 82 e3504–e3515. 10.1128/AEM.03504-15 26826232 PMC4807529

[B23] Hur G. H. Vickery C. R. Burkart M. D. (2012). Explorations of catalytic domains in non-ribosomal peptide synthetase enzymology. *Nat. Prod. Rep.* 29 1074–1098. 10.1039/c2np20025b 22802156 PMC4807874

[B24] Imker H. J. Krahn D. Clerc J. Kaiser M. Walsh C. T. (2010). N-acylation during glidobactin biosynthesis by the tridomain nonribosomal peptide synthetase module GlbF. *Chem. Biol.* 17 1077–1083. 10.1016/j.chembiol.2010.08.007 21035730 PMC3062200

[B25] Joshi M. V. Mann S. G. Antelmann H. Widdick D. A. Fyans J. K. Chandra G. (2010). The twin arginine protein transport pathway exports multiple virulence proteins in the plant pathogen *Streptomyces scabies*. *Mol. Microbiol.* 77 252–271. 10.1111/j.1365-2958.2010.07206.x 20487278

[B26] Kaniusaite M. Tailhades J. Kittilä T. Fage C. D. Goode R. J. Schittenhelm R. B. (2020). Understanding the early stages of peptide formation during the biosynthesis of teicoplanin and related glycopeptide antibiotics. *FEBS J.* 10.1111/febs.15350 [Epub ahead of print]. 32359003

[B27] Kieser T. Bibb M. J. Buttner M. J. Chater K. F. (2000). *Practical Streptomyces Genetics*, 2nd Edn Norwich: John Innes Foundation.

[B28] King R. Lawrence C. Gray J. (2001). Herbicidal properties of the thaxtomin group of phytotoxins. *J. Agric. Food Chem.* 49 2298–2301. 10.1021/jf0012998 11368592

[B29] Koivunen M. Marrone P. (2013). *Uses of Thaxtomin and Thaxtomin Compositions as Herbicides*. U.S. Patent No. US8476195 B2.

[B30] Kreitler D. F. Gemmell E. M. Schaffer J. E. Wencewicz T. A. Gulick A. M. (2019). The structural basis of N-acyl-α-amino-β-lactone formation catalyzed by a nonribosomal peptide synthetase. *Nat. Commun.* 10, 1–13. 10.1038/s41467-019-11383-7 31366889 PMC6668435

[B31] Krissinel E. Henrick K. (2007). Inference of macromolecular assemblies from crystalline state. *J. Mol. Biol.* 372 774–797. 10.1016/j.jmb.2007.05.022 17681537

[B32] Kumar S. Stecher G. Li M. Knyaz C. Tamura K. (2018). MEGA X: molecular evolutionary genetics analysis across computing platforms. *Mol. Biol. Evol.* 35 1547–1549. 10.1093/molbev/msy096 29722887 PMC5967553

[B33] Lautru S. Oves-Costales D. Pernodet J. L. Challis G. L. (2007). MbtH-like protein-mediated cross-talk between non-ribosomal peptide antibiotic and siderophore biosynthetic pathways in *Streptomyces coelicolor* M145. *Microbiology* 153 1405–1412. 10.1099/mic.0.2006/003145-0 17464054

[B34] Leep D. Doricchi L. Perez Baz M. J. Millan F. R. Fernandez Chimeno R. I. (2010). *Use of Thaxtomin for Selective Control of Rice and Aquatic Based Weeds.* WO Patent No 2010/121079 A3.

[B35] Letunic I. Bork P. (2007). Interactive tree Of Life (iTOL): an online tool for phylogenetic tree display and annotation. *Bioinformatics* 23 127–128. 10.1093/bioinformatics/btl529 17050570

[B36] Li Y. Liu J. Adekunle D. Bown L. Tahlan K. Bignell D. R. D. (2019a). TxtH is a key component of the thaxtomin biosynthetic machinery in the potato common scab pathogen *Streptomyces scabies*. *Mol. Plant Pathol.* 20, 1379–1393. 10.1111/mpp.12843 31282068 PMC6792134

[B37] Li Y. Liu J. Díaz-Cruz G. Cheng Z. Bignell D. R. D. (2019b). Virulence mechanisms of plant-pathogenic *Streptomyces* species: an updated review. *Microbiology* 165, 1025–1040. 10.1099/mic.0.000818 31162023

[B38] Loria R. Bukhalid R. A. Creath R. A. Leiner R. H. Olivier M. Steffens J. C. (1995). Differential production of thaxtomins by pathogenic *Streptomyces species in vitro*. *Phytopathology.* 85, 537–541 10.1094/Phyto-85-537

[B39] Marahiel M. A. Stachelhaus T. Mootz H. D. (1997). Modular peptide synthetases involved in nonribosomal peptide synthesis. *Chem. Rev.* 97 2651–2674. 10.1021/cr960029e 11851476

[B40] Martínková L. Uhnáková B. Pátek M. Nešvera J. Křen V. (2009). Biodegradation potential of the genus *Rhodococcus*. *Environ. Int.* 35 162–177. 10.1016/j.envint.2008.07.018 18789530

[B41] McMahon M. D. Rush J. S. Thomas M. G. (2012). Analyses of MbtB, MbtE, and MbtF suggest revisions to the mycobactin biosynthesis pathway in *Mycobacterium tuberculosis*. *J. Bacteriol.* 194:e088-12. 10.1128/JB.00088-12 22447909 PMC3370630

[B42] Meuzelaar H. Vreede J. Woutersen S. (2016). Influence of Glu/Arg, Asp/Arg, and Glu/Lys salt bridges on α-helical stability and folding kinetics. *Biophys. J.* 110 2328–2341. 10.1016/j.bpj.2016.04.015 27276251 PMC4906143

[B43] Miller B. R. Drake E. J. Shi C. Aldrich C. C. Gulick A. M. (2016). Structures of a nonribosomal peptide synthetase module bound to MbtH-like proteins support a highly dynamic domain architecture. *J. Biol. Chem.* 291 22559–22571. 10.1074/jbc.M116.746297 27597544 PMC5077193

[B44] Mori S. Green K. D. Choi R. Buchko G. W. Fried M. G. Garneau-Tsodikova S. (2018a). Using MbtH-Like proteins to alter the substrate profile of a nonribosomal peptide adenylation enzyme. *Chembiochem* 19, 2186–2194. 10.1002/cbic.201800240 30134012 PMC6439349

[B45] Mori S. Pang A. H. Lundy T. A. Garzan A. Tsodikov O. V. Garneau-Tsodikova S. (2018b). Structural basis for backbone N-methylation by an interrupted adenylation domain. *Nat. Chem. Biol.* 14 428–430. 10.1038/s41589-018-0014-7 29556104

[B46] Pojer F. Li S. M. Heide L. (2002). Molecular cloning and sequence analysis of the clorobiocin biosynthetic gene cluster: new insights into the biosynthesis of aminocoumarin antibiotics. *Microbiology* 148 3901–3911. 10.1099/00221287-148-12-3901 12480894

[B47] Quadri L. E. N. Sello J. Keating T. A. Weinreb P. H. Walsh C. T. (1998). Identification of a *Mycobacterium tuberculosis* gene cluster encoding the biosynthetic enzymes for assembly of the virulence-conferring siderophore mycobactin. *Chem. Biol.* 5 631–645. 10.1016/S1074-5521(98)90291-5 9831524

[B48] Sambrook, J. F., and Russell, D. W. (2001). *Molecular Cloning: a Laboratory Manual (3-Volume Set).* Cold Spring Harbor, NY: Cold Spring Harbor Laboratory Press.

[B49] Schneider C. A. Rasband W. S. Eliceiri K. W. (2012). NIH Image to ImageJ: 25 years of image analysis. *Nat. Methods* 9 671–675. 10.1038/nmeth.2089 22930834 PMC5554542

[B50] Schomer R. A. Park H. Barkei J. J. Thomas M. G. (2018). Alanine scanning of YbdZ, an MbtH-like protein, reveals essential residues for functional interactions with its nonribosomal peptide synthetase partner EntF. *Biochemistry* 57, 4125–4134. 10.1021/acs.biochem.8b00552 29921120 PMC6050124

[B51] Schomer R. A. Thomas M. G. (2017). Characterization of the functional variance in MbtH-like protein interactions with a nonribosomal peptide synthetase. *Biochemistry* 56 5380–5390. 10.1021/acs.biochem.7b00517 28880538 PMC5902190

[B52] Strieker M. Tanović A. Marahiel M. A. (2010). Nonribosomal peptide synthetases: structures and dynamics. *Curr. Opin. Struct. Biol.* 20 234–240. 10.1016/j.sbi.2010.01.009 20153164

[B53] Süssmuth R. D. Mainz A. (2017). Nonribosomal peptide synthesis-principles and prospects. *Angew. Chem.* 56 3770–3821. 10.1002/anie.201609079 28323366

[B54] Tarry M. J. Haque A. S. Bui K. H. Schmeing T. M. (2017). X-Ray crystallography and electron microscopy of cross- and multi-module nonribosomal peptide synthetase proteins reveal a flexible architecture. *Structure* 25 89–96. 10.1016/j.str.2017.03.014 28434915

[B55] Waterhouse A. Bertoni M. Bienert S. Studer G. Tauriello G. Gumienny R. (2018). SWISS-MODEL: homology modelling of protein structures and complexes. *Nucleic Acids Res. Spec. Publ.* 46:gky427. 10.1093/nar/gky427 29788355 PMC6030848

[B56] Whelan S. Goldman N. (2001). A general empirical model of protein evolution derived from multiple protein families using a maximum-likelihood approach. *Mol. Biol. Evol.* 18 691–699. 10.1093/oxfordjournals.molbev.a003851 11319253

[B57] Wolpert M. Gust B. Kammerer B. Heide L. (2007). Effects of deletions of mbtH-like genes on clorobiocin biosythesis in *Streptomyces coelicolor*. *Microbiology* 153, 1413–1423. 10.1099/mic.0.2006/002998-0 17464055

[B58] Xie H. Vucetic S. Iakoucheva L. M. Oldfield C. J. Dunker A. K. Obradovic Z. (2007). Functional anthology of intrinsic disorder. 3. Ligands, post-translational modifications, and diseases associated with intrinsically disordered proteins. *Proteome Res.* 6 1917–1932. 10.1021/pr060394e 17391016 PMC2588348

[B59] Zhang W. Heemstra J. R. Walsh C. T. Imker H. J. (2010). Activation of the pacidamycin pacl adenylation domain by MbtH-like proteins. *Biochemistry* 49 9946–9947. 10.1021/bi101539b 20964365 PMC2982891

[B60] Zhang Y. Bignell D. R. Zuo R. Fan Q. Huguet-Tapia J. C. Ding Y. (2016). Promiscuous pathogenicity islands and phylogeny of pathogenic *Streptomyces* spp. *Mol. Plant Microbe Interact.* 29 640–650. 10.1094/MPMI-04-16-0068-R 27502745

[B61] Zolova O. E. Garneau-Tsodikova S. (2012). Importance of the MbtH-like protein TioT for production and activation of the thiocoraline adenylation domain of TioK. *Medchemcomm* 3 950–955. 10.1039/c2md20131c

[B62] Zolova O. E. Garneau-Tsodikova S. (2014). KtzJ-dependent serine activation and O-methylation by KtzH for kutznerides biosynthesis. *J. Antibiot.* 67:ja.2013.98. 10.1038/ja.2013.98 24105608

[B63] Zwahlen R. D. Pohl C. Bovenberg R. A. L. Driessen A. J. M. (2019). Bacterial MbtH-like proteins stimulate nonribosomal peptide synthetase-derived secondary metabolism in filamentous fungi. *ACS Synth. Biol.* 8 1776–1787. 10.1021/acssynbio.9b00106 31284717 PMC6713467

