# Supplementary material for: Functional Cross-Talk of MbtH-Like Proteins During Thaxtomin Biosynthesis in the Potato Common Scab Pathogen Streptomyces scabiei
Source: Front Microbiol. 2020 Oct 15;11:585456. doi: 10.3389/fmicb.2020.585456 (PMC7593251; doi:10.3389/fmicb.2020.585456)
Supplement: Supplementary file 1 [file Image_1.PDF]

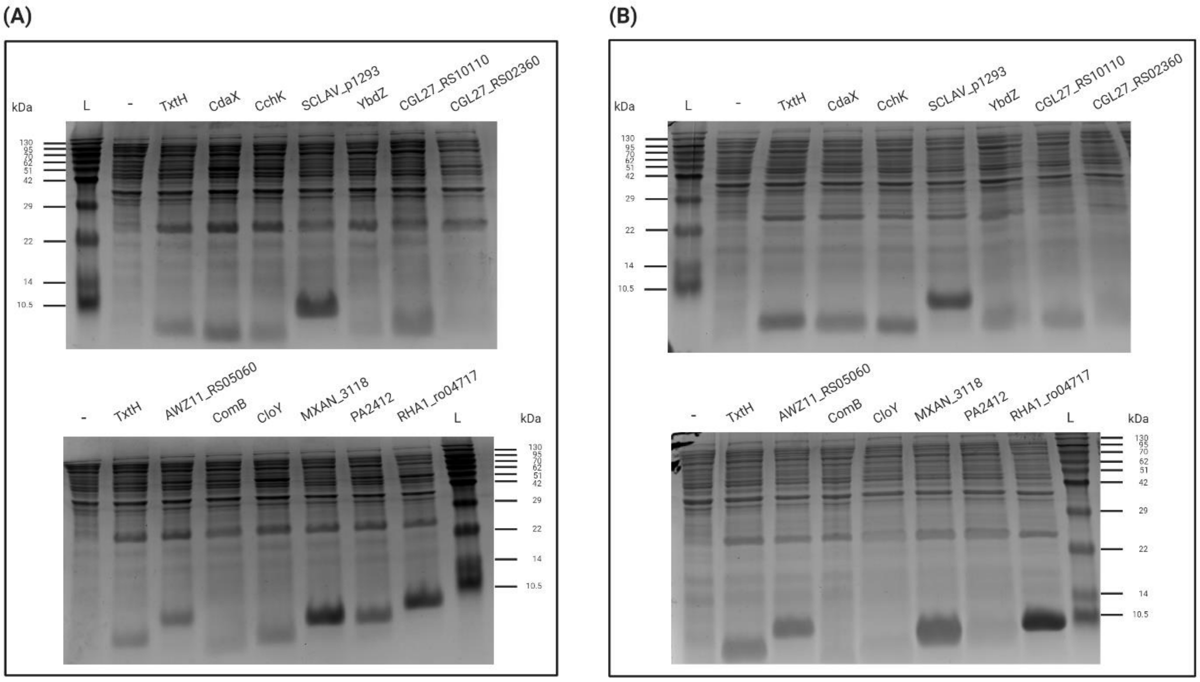

Supplementary Figure 1. SDS-PAGE analysis of total soluble protein extracts from *E. coli* BL21(DE3)*ybdZ:aac(3)IV* expressing HIS$_6$-TxtA^A (A) or HIS$_6$-TxtB^A (B) in the presence and absence (-) of different HIS$_6$-tagged MLPs. Lane L: Pink Plus Prestained Protein Ladder (FroggaBio Inc). The MLPs are visible as prominent bands below the 10.5 kDa marker band.
