# Supplementary material for: Functional Cross-Talk of MbtH-Like Proteins During Thaxtomin Biosynthesis in the Potato Common Scab Pathogen Streptomyces scabiei
Source: Front Microbiol. 2020 Oct 15;11:585456. doi: 10.3389/fmicb.2020.585456 (PMC7593251; doi:10.3389/fmicb.2020.585456)
Supplement: Supplementary file 2 [file Image_2.PDF]

(A)

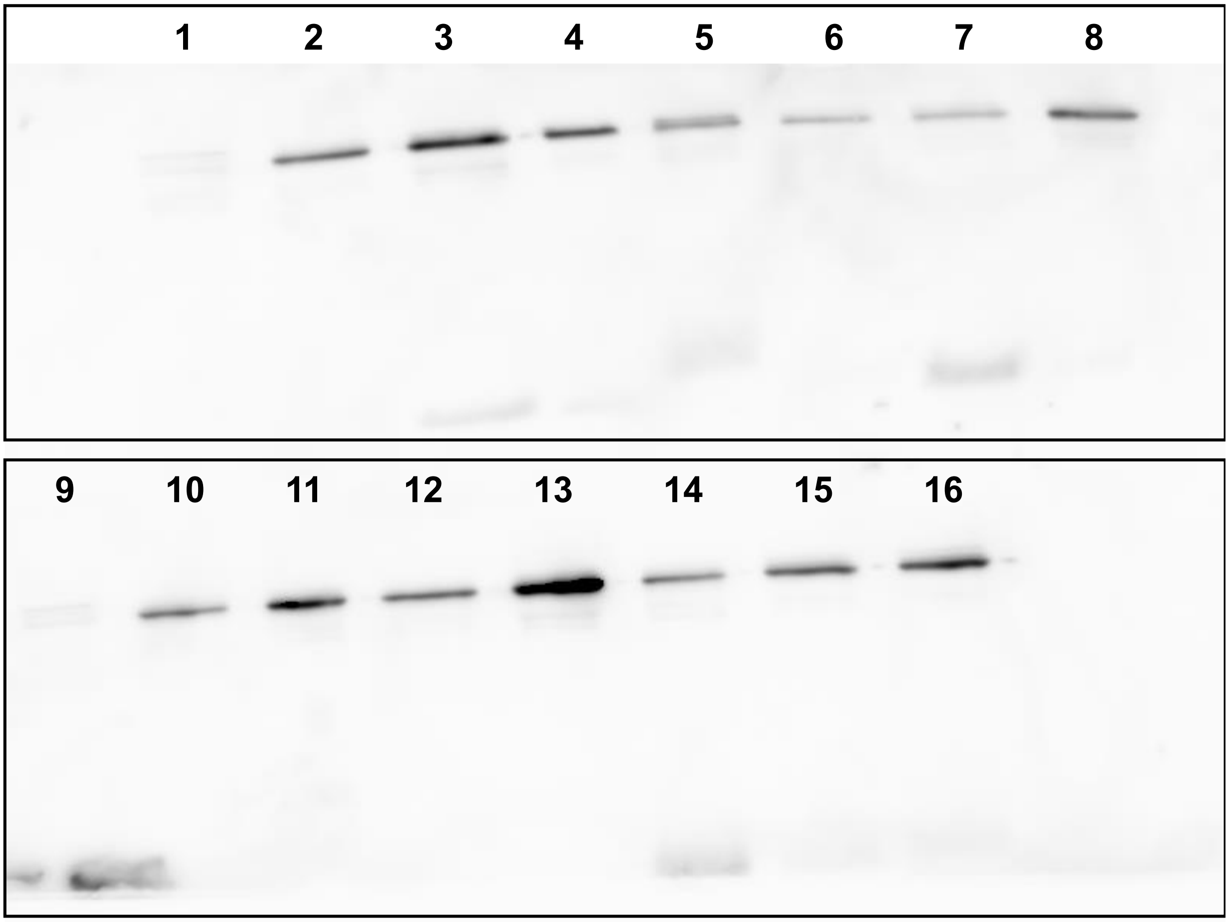

(B)

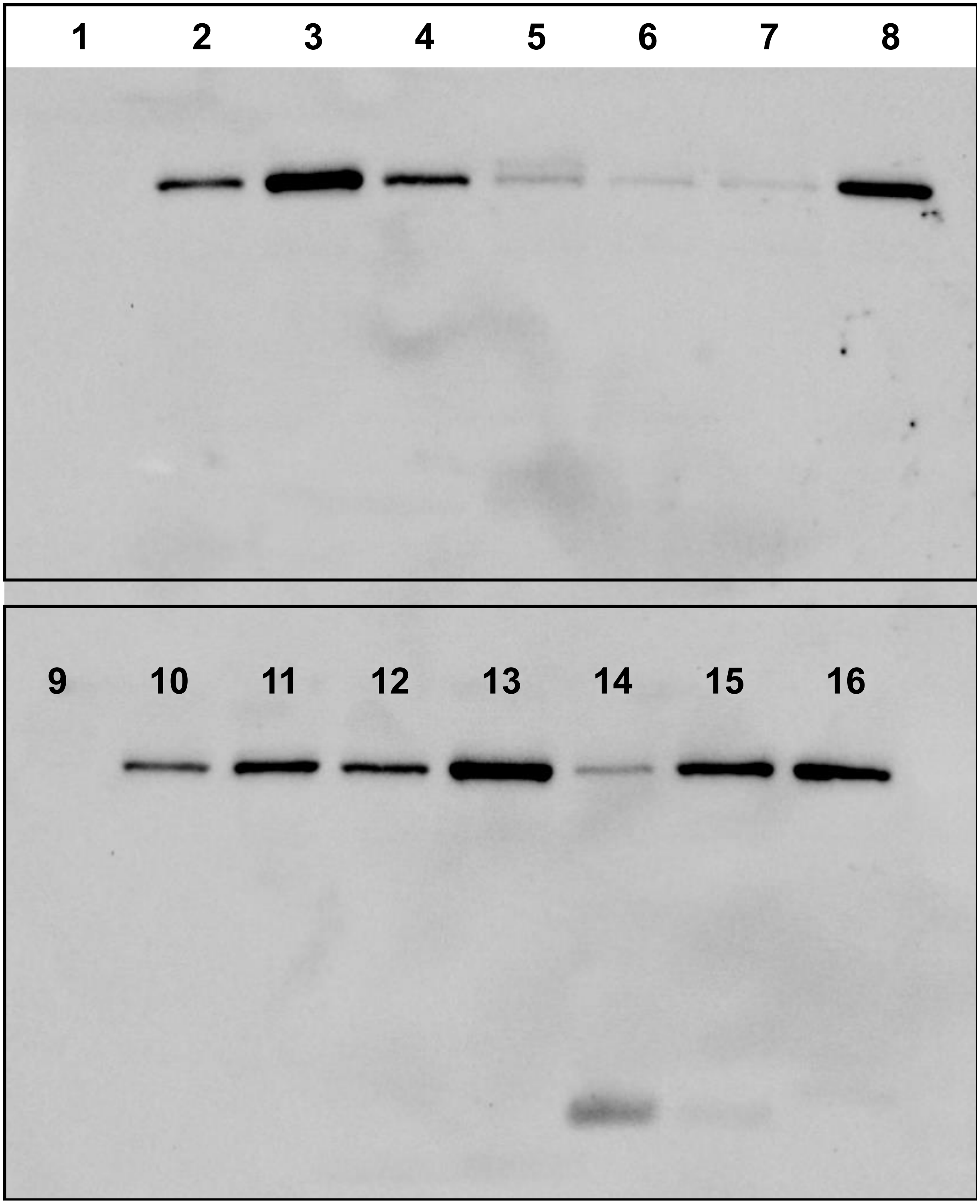

(C)

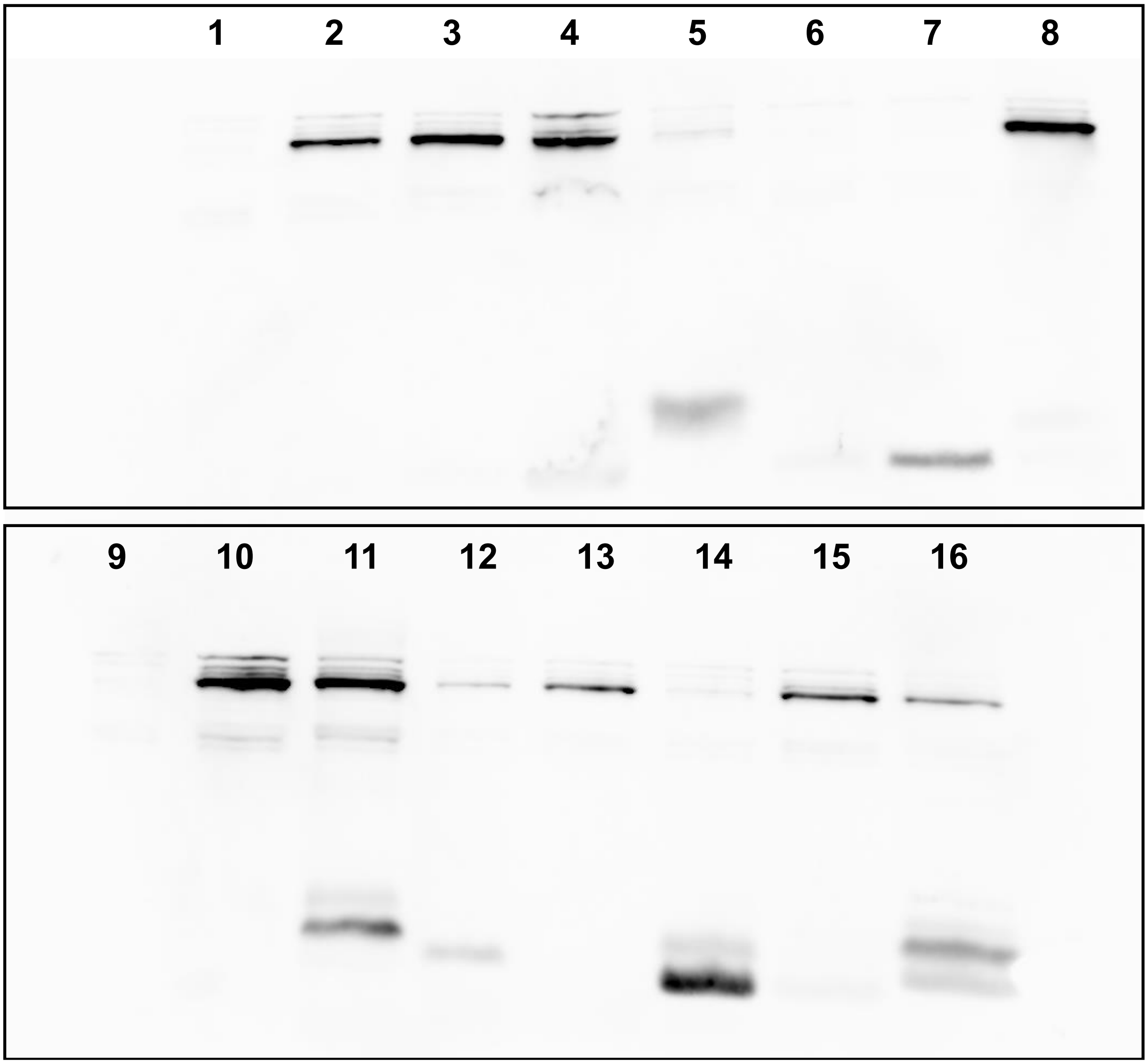

(D)

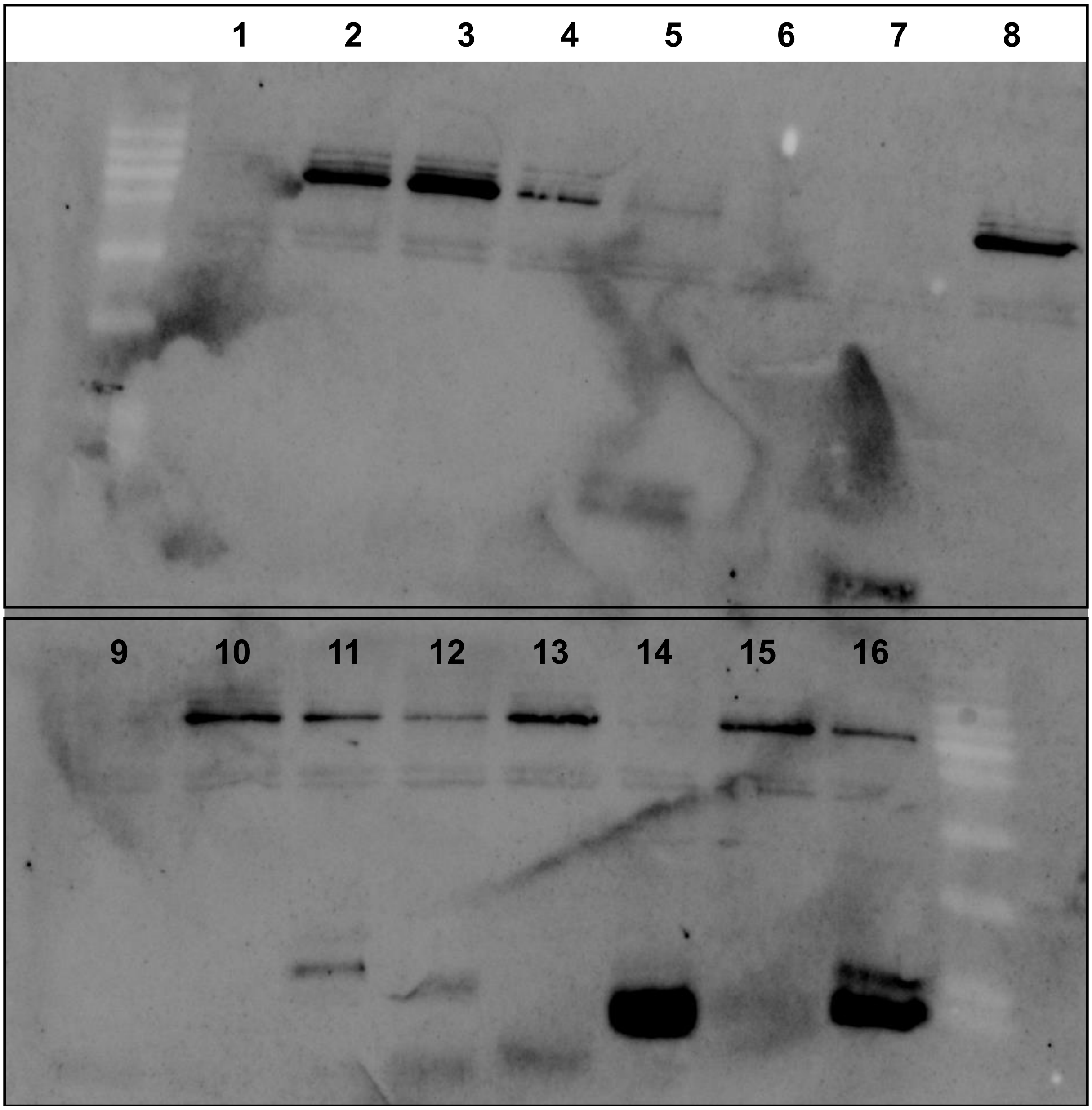

(E)

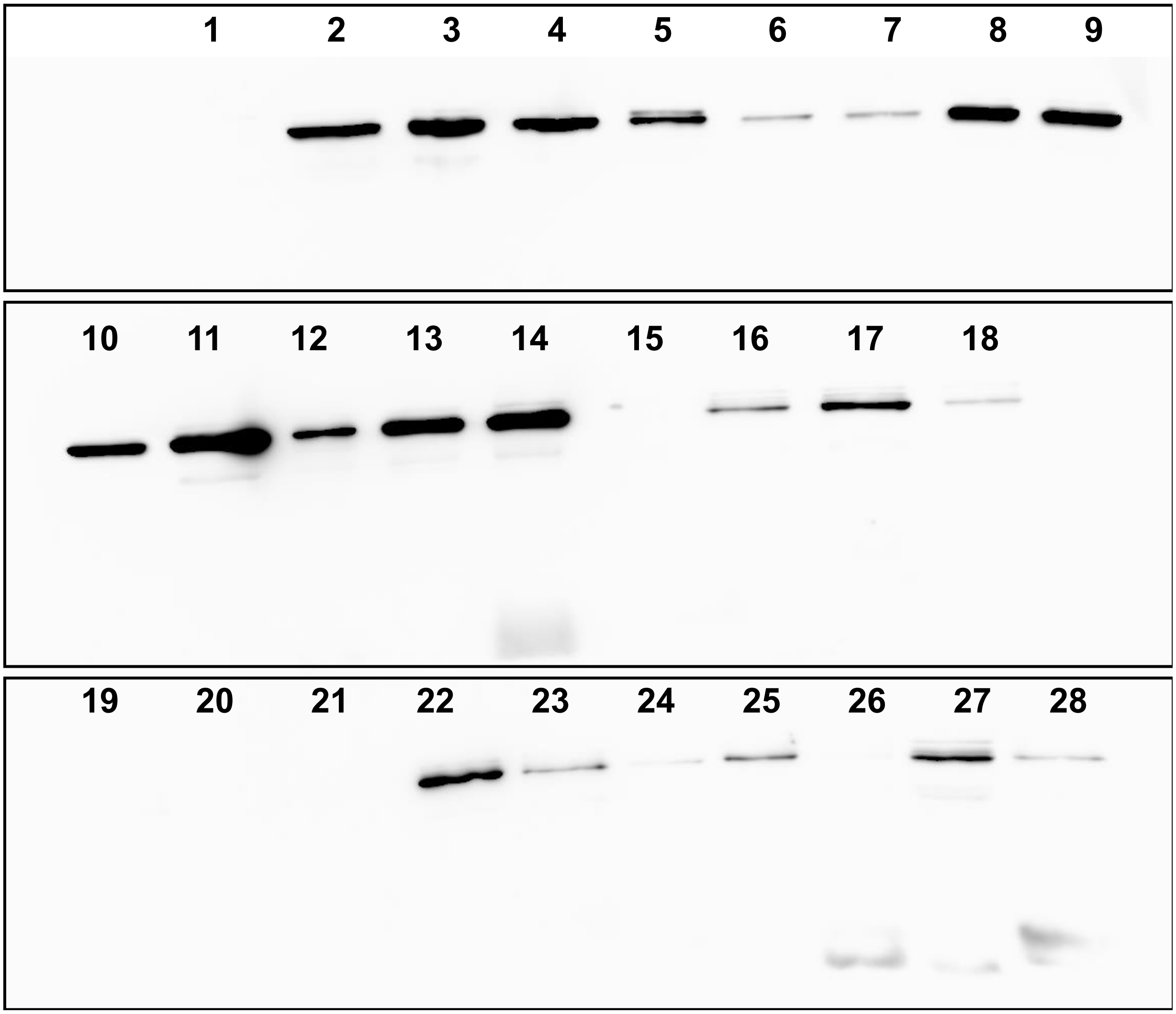

Supplementary Figure 2. Western blot analysis of soluble HIS_6-TxtA^A and HIS_6-TxtB^A proteins expressed in the presence and absence of different HIS_6-tagged MLPs. (A, B) Duplicate membranes of soluble HIS_6-TxtA^A expressed in the absence of an MLP (lanes 1 and 9) or co-expressed with HIS_6-TxtH (lanes 2 and 10), HIS_6-CdaX (lane 3), HIS_6-CchK (lane 4), HIS_6-SCLAV_p1293 (lane 5), HIS_6-YbdZ (lane 6), HIS_6-CGL27_RS10110 (lane 7), HIS_6-CGL27_RS02360 (lane 8), HIS_6-AWZ11_RS05060 (lane 11), HIS_6-ComB (lane 12), HIS_6-CloY (lane 13), HIS_6-MXAN_3118 (lane 14), HIS_6-PA2412 (lane 15), and HIS_6-RHA1_ro04717 (lane 16). (C, D) Duplicate membranes of soluble HIS_6-TxtB^A expressed in the absence of an MLP (lanes 1 and 9) or co-expressed with HIS_6-TxtH (lanes 2 and 10), HIS_6-CdaX (lane 3), HIS_6-CchK (lane 4), HIS_6-SCLAV_p1293 (lane 5), HIS_6-YbdZ (lane 6), HIS_6-CGL27_RS10110 (lane 7), HIS_6-CGL27_RS02360 (lane 8), HIS_6-AWZ11_RS05060 (lane 11), HIS_6-ComB (lane 12), HIS_6-CloY (lane 13), HIS_6-MXAN_3118 (lane 14), HIS_6-PA2412 (lane 15), and HIS_6-RHA1_ro04717 (lane 16). (E) Third replicate set of membranes of soluble HIS_6-TxtA^A and HIS_6-TxtB^A expressed in the presence and absence of different MLPs. HIS_6-TxtA^A expressed without an MLP (lane 1) or co-expressed with HIS_6-TxtH (lane 2), HIS_6-CdaX (lane 3), HIS_6-CchK (lane 4), HIS_6-

SCLAV_p1293 (lane 5), HIS_6-YbdZ (lane 6), HIS_6-CGL27_RS10110 (lane 7), HIS_6-CGL27_RS02360 (lane 8), HIS_6-AWZ11_RS05060 (lane 9), HIS_6-ComB (lane 10), HIS_6-CloY (lane 11), HIS_6- MXAN_3118 (lane 12), HIS_6-PA2412 (lane 13), and HIS_6-RHA1_ro04717 (lane 14). HIS_6-TxtB^A expressed without an MLP (lane 15) or co-expressed with HIS_6-TxtH (lane 16), HIS_6-CdaX (lane 17), HIS_6-CchK (lane 18), HIS_6-SCLAV_p1293 (lane 19), HIS_6-YbdZ (lane 20), HIS_6-CGL27_RS10110 (lane 21), HIS_6-CGL27_RS02360 (lane 22), HIS_6-AWZ11_RS05060 (lane 23), HIS_6-ComB (lane 24), HIS_6-CloY (lane 25), HIS_6-MXAN_3118 (lane 26), HIS_6-PA2412 (lane 27), and HIS_6-RHA1_ro04717 (lane 28).
