# Supplementary material for: Functional Cross-Talk of MbtH-Like Proteins During Thaxtomin Biosynthesis in the Potato Common Scab Pathogen Streptomyces scabiei
Source: Front Microbiol. 2020 Oct 15;11:585456. doi: 10.3389/fmicb.2020.585456 (PMC7593251; doi:10.3389/fmicb.2020.585456)
Supplement: Supplementary file 3 [file Image_3.PDF]

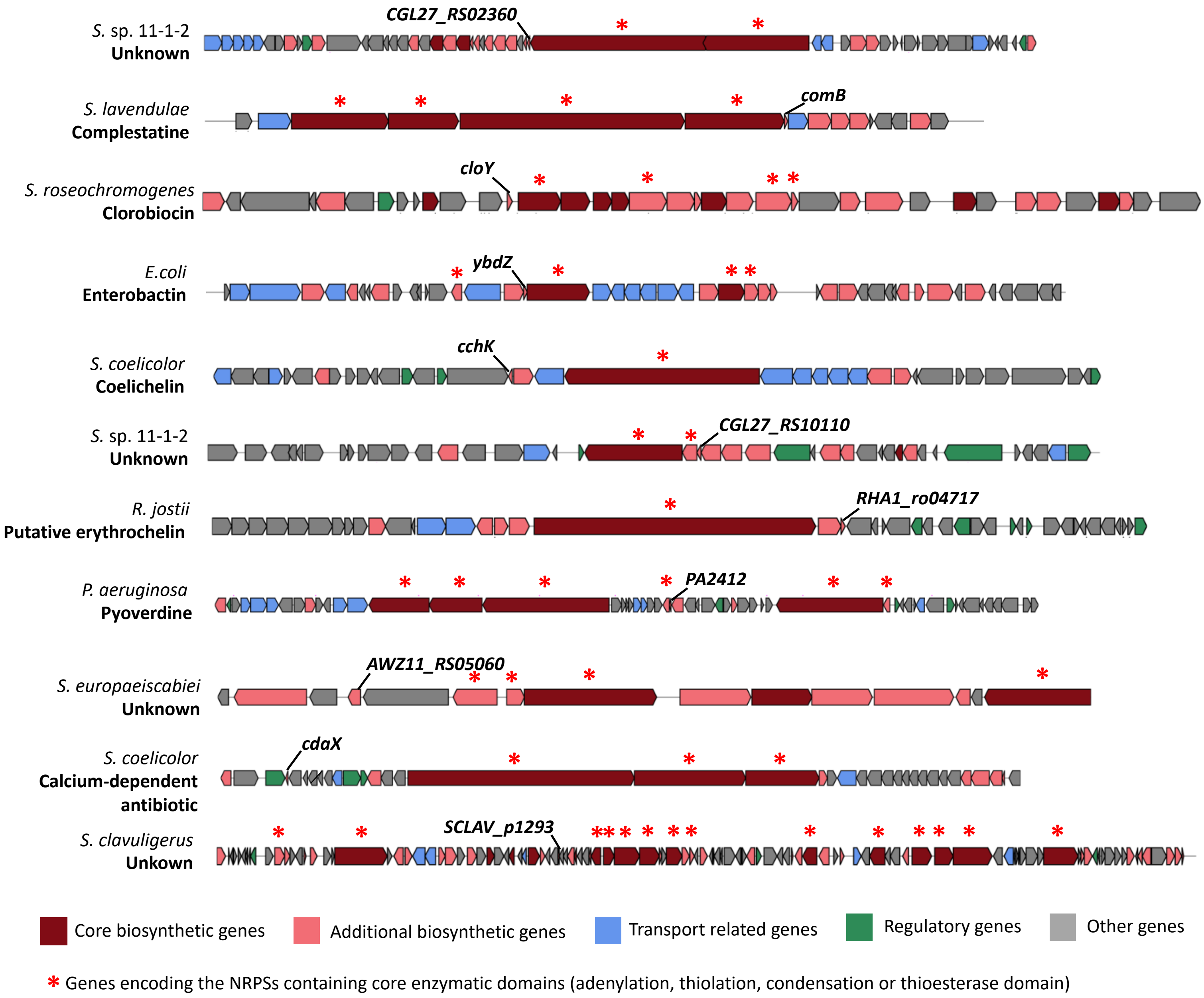

S. sp. 11-1-2
Unknown
CGL27_RS02360
S. lavendulae
Complestatine
comB
S. roseochromogenes
Clorobiocin
cloY
E.coli
Enterobactin
ybdZ
S. coelicolor
Coelichelin
cchK
S. sp. 11-1-2
Unknown
CGL27_RS10110
R. jostii
Putative erythrochelin
RHA1_ro04717
P. aeruginosa
Pyoverdine
PA2412
S. europaeiscabiei
Unknown
AWZ11_RS05060
S. coelicolor
Calcium-dependent antibiotic
cdaX
S. clavuligerus
Unkown
SCLAV_p1293
Core biosynthetic genes
Additional biosynthetic genes
Transport related genes
Regulatory genes
Other genes
* Genes encoding the NRPSs containing core enzymatic domains (adenylation, thiolation, condensation or thioesterase domain)

Supplementary Figure 3. Known or predicted non-ribosomal peptide biosynthetic gene clusters harbouring the MbtH-like protein (MLP)-coding genes used in this study.
