# Supplementary material for: Functional Cross-Talk of MbtH-Like Proteins During Thaxtomin Biosynthesis in the Potato Common Scab Pathogen Streptomyces scabiei
Source: Front Microbiol. 2020 Oct 15;11:585456. doi: 10.3389/fmicb.2020.585456 (PMC7593251; doi:10.3389/fmicb.2020.585456)
Supplement: Supplementary file 4 [file Image_4.PDF]

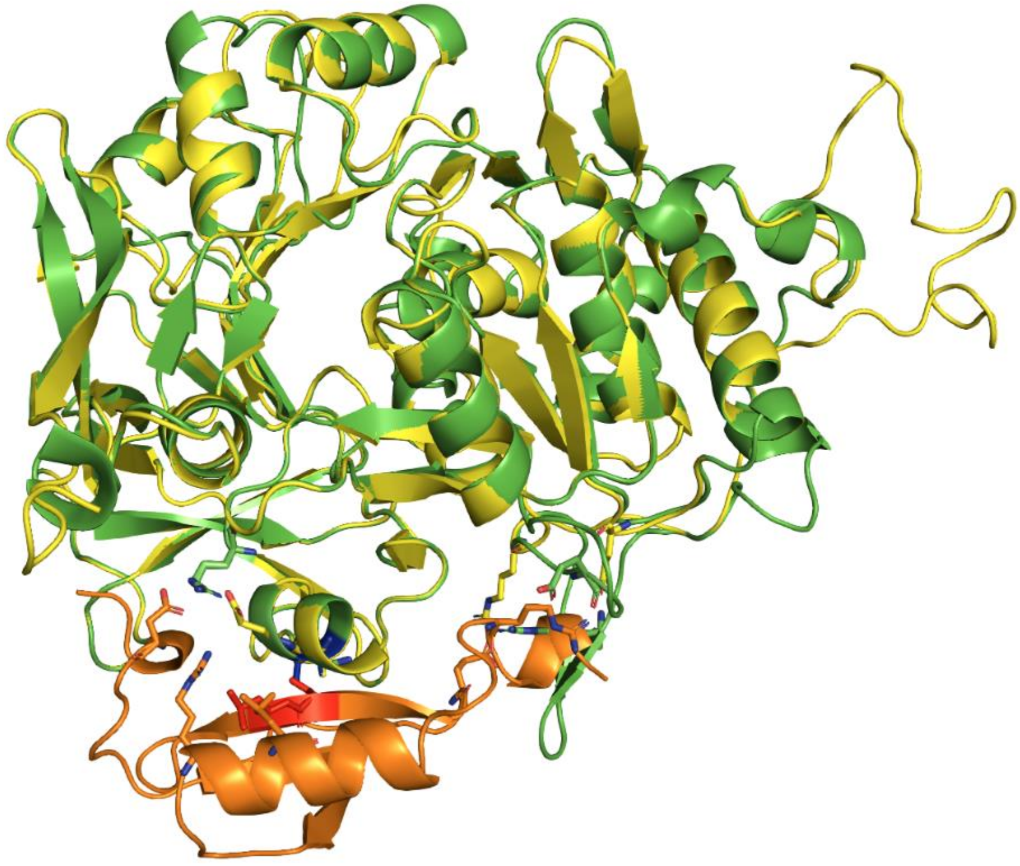

Supplementary Figure 4. Predicted 3-dimentional structures of the *S. scabiei* $TxtA^A$ (green), $TxtB^A$ (yellow) and TxtH (orange). The A-domain structures were predicted using the crystal structure of TioS from *Micromonospora* sp. ML1 (PDB: 5wmm_1) as the template, and the structure of the TxtH MLP was predicted using the crystal structure of FscK from *Thermobifida fusca* (PDB: 6ea3_1) as the template. The generated model of TxtH positioned next to the A-domains is based on the location of the TioT MLP that is bound to the A-domain in TioS. A more detailed image of the predicted interaction interface is shown in Figure 6A.
