# Supplementary material for: Functional Cross-Talk of MbtH-Like Proteins During Thaxtomin Biosynthesis in the Potato Common Scab Pathogen Streptomyces scabiei
Source: Front Microbiol. 2020 Oct 15;11:585456. doi: 10.3389/fmicb.2020.585456 (PMC7593251; doi:10.3389/fmicb.2020.585456)
Supplement: Supplementary file 5 [file Data_Sheet_1.DOCX]

**Supplementary Data 1.** Nucleotide sequences of the DNA fragments synthesized by TWIST BIOSCIENCE. The MLP coding sequence within each fragment is indicated in blue. Coding sequences that were codon-optimized for expression in *Streptomyces* are indicated with *.

*Myxococcus xanthus* DK1622 *MXAN_3118**

TGTCTTGACCCCTTTTCCTGGATTGTGGCCAGAATACGCCCCGTTCCAGGAGAGAAATCA**ATGACCGACGAACGGGAGGACACCACCGTCTACAAGGTGGTGGTGAACCACGAAGAACAGTACTCCATCTGGCCGGCGGACCGCGAAAACGCGCTCGGGTGGAAGGACGCGGGCAAGCAGGGCCTCAAGGCGGAGTGCCTGGAATACATCAAGGAAGTCTGGACCGACATGCGCCCGCTCAGCCTGCGCAAGAAGATGGAAGAACTCAAGAGCTAG**TCACGGCACCGTCCGTGCGCACGCCCCTCG

*Rhodococcus jostii* RHA1 *RHA1_ro04717**

CCGCAACGGCACGCTTGCTAGTGTGAACGCACAGGACACGTATGCGACAAGCGAGGCGAG**ATGTCCACCAACCCCTTCGACGACGAAGAGGGGCGGTTCTACGTCCTGGTCAACGACGAGGACCAGCACTCCCTGTGGCCCACGTTCTCCGAGGTCCCCGCGGGGTGGCGGGTCGTGTTCGGCGAAGAGTCGCGCGCCGCGTGCCTCGAATACGTCGAAAAGAACTGGACCGACATGCGCCCCAAGAGCCTCCGCGAAGCGATGGAAGCCGACGAAAAGTCCGGCGGGCGGCACAGCGTCGACAAGAGCTGA**CCTCCCCGGCGCCGCGTTTCGGCGACGATG

*Streptomyces roseochromogenes* subsp. *oscitans* DS12.976 *cloY*

CACTCCGGGCGGTCAATGGTGGAACAAGACGCGTGAAATAACATCTGGGAGGTATTCGTC**ATGGCGACGAACCCGTTCGAGGACGAGAACGGCTCCTATCTGGTCCTGATCAATGGCGAGGGGCAGCATTCTCTGTGGCCTTCGTTCGCTGATGTTCCCAACGGGTGGACTGTCATTTTCAACGAGGCGTCGCGGCAAGACTGCCTCGATTACGTCAATGAGCATTGGACAGACATGCGGCCGTTGAGCCTGCAGCGGGCGATGGGTGGCGAGTAG**CATCTGCTCATTCAAAAGTG GATTTGGCAA

*Streptomyces lavendulae* *comB*

CCGCCCCTCGACAGACCGAAGAATCCGCGAACCACTCCGGAAAAGAAGGTGAATGACACC**ATGACTAACCCTTTCGACAACGAGAACGGCACTTTCCTGGTGCTCGTCAACGACGAGGGTCAGCACTCGCTCTGGCCGGTTTTCGCGGAGATCCCGCAGGGCTGGACGACCGCGTTCGGTGAGGCGAGCCGGGCCGAATGCCTGGAATTCGTCGAGCAGAACTGGACCGACATGCGGCCCAAGAGCCTCGTCGCCCGTATGGAGGGCACCGCCACGGCCTGA**GAGATTGAGCACTCACGCATTGAGAAGAAG

*Pseudomonas aeruginosa* PA01 *PA2412**

TCGTTATCCAACGCAAGGGCCGTGCTGCGCGCGGCCGTTCGATCCCATCAGGAGCAAGCA**ATGACTTCAGTGTTCGACCGTGACGACATCCAGTTCCAGGTAGTGGTCAACCATGAGGAGCAGTATTCCATCTGGCCGGAATACAAGGAGATTCCCCAGGGCTGGCGGGCGGCCGGCAAGAGCGGCCTGAAGAAGGACTGCCTGGCCTACATCGAGGAAGTCTGGACCGACATGCGCCCGCTGAGCCTGCGCCAGCACATGGACAAGGCGGCCGGCTGA**GTGGGCGGTACGCCCGTACGGCTGTTCTGC

*Escherichia coli* BL21(DE3) *ybdZ**

GGGGCTAATCGACCTCTGGCAACCACTTTTCCATGACAGGAGTTGAAT**ATGGCCTTCTCCAACCCCTTCGACGACCCCCAGGGGGCCTTCTACATCCTCCGGAACGCCCAGGGCCAGTTCTCCCTCTGGCCCCAGCAGTGCGTGCTCCCGGCGGGCTGGGACATCGTGTGCCAGCCGCAGAGCCAGGCCTCGTGCCAGCAGTGGCTCGAAGCGCACTGGCGGACGCTGACCCCGACGAACTTCACCCAGCTCCAGGAAGCGCAGTGA**GCCAGCATTTACCTTTGGTCGCCGCACAGCCCG
