# Supplementary material for: Functional Cross-Talk of MbtH-Like Proteins During Thaxtomin Biosynthesis in the Potato Common Scab Pathogen Streptomyces scabiei
Source: Front Microbiol. 2020 Oct 15;11:585456. doi: 10.3389/fmicb.2020.585456 (PMC7593251; doi:10.3389/fmicb.2020.585456)
Supplement: Supplementary file 7 [file Table_1.DOCX]

**Supplementary Table 1.** Oligonucleotide primers used in this study.

| Primer | Sequence (5′ - 3′)† | Use |
| --- | --- | --- |
| PL169 | GCGC**CATATG**ACCAATCCGTTCGAAGACGC | Forward primer for construction of pET28b/HIS_6_-*cdaX* |
| PL170 | GCGC**GAATTC**TCAGTTGCCGGTGCTCATCG | Reverse primer for construction of pET28b/HIS_6_-*cdaX* |
| PL183 | GCGC**CATATG**AGCACCAACCCCTTCGACGA | Forward primer for construction of pET28b/HIS_6_-*cchK* |
| PL184 | GCGC**GAATTC**TCAGGCGTCCGCGGTCCGGG | Reverse primer for construction of pET28b/HIS_6_-*cchK* |
| PL167 | GCGC**CATATG**AGCGGCGATGTGCGGGAGCG | Forward primer for construction of pET28b/HIS_6_-*SCLAV_p1293* |
| PL168 | GCGC**GAATTC**TCACCGGGCCTCCGCCTCCG | Reverse primer for construction of pET28b/HIS_6_-*SCLAV_p1293* |
| PL175 | GCGC**CATATG**AGCGCCTCACCCGCCCTGCG | Forward primer for construction of pET28b/HIS_6_-*CGL27_RS10110* |
| PL176 | GCGC**GAATTC**TCATCGTGAGGCTCGTACGGA | Reverse primer for construction of pET28b/HIS_6_-*CGL27_RS10110* |
| PL177 | GCGC**CATATG**AGCAACCCCTTCGACGACGC | Forward primer for construction of pET28b/HIS_6_-*CGL27_RS02360* |
| PL178 | GCGC**GAATTC**TCAGGAGGCGGCCGCGTCCA | Reverse primer for construction of pET28b/HIS_6_-*CGL27_RS02360* |
| PL173 | GCGC**CATATG**GCAGTGAACCCGTTCGACGA | Forward primer for construction of pET28b/HIS_6_-*AWZ11_RS05060* |
| PL174 | GCGC**GAATTC**TCAGGGCGCGGGGGTCGCCT | Reverse primer for construction of pET28b/HIS_6_-*AWZ11_RS05060* |
| PL185 | GCGC**CATATG**ACCGACGAACGGGAGGACAC | Forward primer for construction of pET28b/HIS_6_-MXAN_3118 |
| PL186 | GCGC**GAATTC**CTAGCTCTTGAGTTCTTCCA | Reverse primer for construction of pET28b/HIS_6_-MXAN_3118 |
| PL187 | GCGC**CATATG**TCCACCAACCCCTTCGACGA | Forward primer for construction of pET28b/HIS_6_-RHA1_ro04717 |
| PL188 | GCGC**GAATTC**TCAGCTCTTGTCGACGCTGT | Reverse primer for construction of pET28b/HIS_6_-RHA1_ro04717 |
| PL189 | GCGC**CATATG**GCGACGAACCCGTTCGAGGA | Forward primer for construction of pET28b/HIS_6_-*cloY* |
| PL190 | GCGC**GAATTC**CTACTCGCCACCCATCGCCC | Reverse primer for construction of pET28b/HIS_6_-*cloY* |
| PL191 | GCGC**CATATG**ACTAACCCTTTCGACAACGA | Forward primer for construction of pET28b/HIS_6_-*comB* |
| PL192 | GCGC**GGATCC**TCAGGCCGTGGCGGTGCCCT | Reverse primer for construction of pET28b/HIS_6_-*comB* |
| PL193 | GCGC**CATATG**ACTTCAGTGTTCGACCGTGA | Forward primer for construction of pET28b/HIS_6_-*PA2412* |
| PL194 | GCGC**GAATTC**TCAGCCGGCCGCCTTGTCCA | Reverse primer for construction of pET28b/HIS_6_-*PA2412* |
| PL208 | GCGC**CATATG**GCCTTCTCCAACCCCTTCGA | Forward primer for construction of pET28b/HIS_6_-*ybdZ* |
| PL209 | ATAT**GCGGCCGC**TCACTGCGCTTCCTGGAGCT | Reverse primer for construction of pET28b/HIS_6_-*ybdZ* |
| PL35 | GCGC**CATATG**CCCTCACCCTTCGACGAC | Forward primer for construction of pRFSRL16/*txtH* |
| PL195 | ATAT**GCGGCCGC**TCATTCACGGACGGACGCCG | Reverse primer for construction of pRFSRL16/*txtH* |
| PL163 | GCGC**CATATG**ACCAACCCCTTCGAGAAC | Forward primer for construction of pRFSRL16/*mlp_lipo_* |
| PL196 | ATAT**GCGGCCGC**TCACTCGCCCATGGCCCGGA | Reverse primer for construction of pRFSRL16/*mlp_lipo_* |
| PL169 | GCGC**CATATG**ACCAATCCGTTCGAAGACGC | Forward primer for construction of pRFSRL16/*cdaX* |
| PL198 | ATAT**GCGGCCGC**TCAGTTGCCGGTGCTCATCG | Reverse primer for construction of pRFSRL16/*cdaX* |
| PL167 | GCGC**CATATG**AGCGGCGATGTGCGGGAGCG | Forward primer for construction of pRFSRL16/*SCLAV_p1293* |
| PL199 | ATAT**GCGGCCGC**TCACCGGGCCTCCGCCTCCG | Reverse primer for construction of pRFSRL16/*SCLAV_p1293* |
| PL175 | GCGC**CATATG**AGCGCCTCACCCGCCCTGCG | Forward primer for construction of pRFSRL16/*CGL27_RS10110* |
| PL200 | ATAT**GCGGCCGC**TCATCGTGAGGCTCGTACGGA | Reverse primer for construction of pRFSRL16/*CGL27_RS10110* |
| PL177 | GCGC**CATATG**AGCAACCCCTTCGACGACGC | Forward primer for construction of pRFSRL16/*CGL27_RS02360* |
| PL214 | ATAT**GCGGCCGC**TCAGGAGGCTGCCGCGTCCATGGCCTCGAC | Reverse primer for construction of pRFSRL16/*CGL27_RS02360* |
| PL173 | GCGC**CATATG**GCAGTGAACCCGTTCGACGA | Forward primer for construction of pRFSRL16/*AWZ11_RS05060* |
| PL201 | ATAT**GCGGCCGC**TCAGGGCGCGGGGGTCGCCT | Reverse primer for construction of pRFSRL16/*AWZ11_RS05060* |
| PL183 | GCGC**CATATG**AGCACCAACCCCTTCGACGA | Forward primer for construction of pRFSRL16/*cchK* |
| PL202 | ATAT**GCGGCCGC**TCAGGCGTCCGCGGTCCGGG | Reverse primer for construction of pRFSRL16/*cchK* |
| PL185 | GCGC**CATATG**ACCGACGAACGGGAGGACAC | Forward primer for construction of pRFSRL16/*MXAN3118* |
| PL203 | ATAT**GCGGCCGC**CTAGCTCTTGAGTTCTTCCA | Reverse primer for construction of pRFSRL16/*MXAN3118* |
| PL187 | GCGC**CATATG**TCCACCAACCCCTTCGACGA | Forward primer for construction of pRFSRL16/*RHA1_ro04717* |
| PL204 | ATAT**GCGGCCGC**TCAGCTCTTGTCGACGCTGT | Reverse primer for construction of pRFSRL16/*RHA1_ro04717* |
| PL189 | GCGC**CATATG**GCGACGAACCCGTTCGAGGA | Forward primer for construction of pRFSRL16/*cloY* |
| PL205 | ATAT**GCGGCCGC**CTACTCGCCACCCATCGCCC | Reverse primer for construction of pRFSRL16/*cloY* |
| PL191 | GCGC**CATATG**ACTAACCCTTTCGACAACGA | Forward primer for construction of pRFSRL16/*comB* |
| PL206 | ATAT**GCGGCCGC**TCAGGCCGTGGCGGTGCCCT | Reverse primer for construction of pRFSRL16/*comB* |
| PL193 | GCGC**CATATG**ACTTCAGTGTTCGACCGTGA | Forward primer for construction of pRFSRL16/*PA2412* |
| PL207 | ATAT**GCGGCCGC**TCAGCCGGCCGCCTTGTCCA | Reverse primer for construction of pRFSRL16/*PA2412* |
| PL208 | GCGC**CATATG**GCCTTCTCCAACCCCTTCGA | Forward primer for construction of pRFSRL16/*ybdZ* |
| PL209 | ATAT**GCGGCCGC**TCACTGCGCTTCCTGGAGCT | Reverse primer for construction of pRFSRL16/*ybdZ* |

† Non-homologous extensions are underlined, while engineered restriction sites are indicated in bold.
