# Supplementary material for: Functional Cross-Talk of MbtH-Like Proteins During Thaxtomin Biosynthesis in the Potato Common Scab Pathogen Streptomyces scabiei
Source: Front Microbiol. 2020 Oct 15;11:585456. doi: 10.3389/fmicb.2020.585456 (PMC7593251; doi:10.3389/fmicb.2020.585456)
Supplement: Supplementary file 9 [file Table_3.docx]

**Supplementary Table** **3.** Quality parameters of structural models built for TxtA^A^, TxtB^A^ and TxtH using SwissModel.

| Protein | TxtA^A^ | TxtB^A^ | TxtH |
| --- | --- | --- | --- |
| Template PDB | 5wmm_1 | 5wmm_1 | 6ea3_1 |
| Description | TioS NRPS from *Micromonospora*  sp. ML1 | TioS NRPS from *Micromonospora*  sp. ML1 | FscK MLP from *Thermobifida fusca* |
| Reference | Mori et al., 2018a | Mori et al., 2018a | Bruner and Zagulyaeva, unpublished |
| Method | X-ray, 2.9Å | X-ray, 2.9Å | X-ray, 1.65Å |
| Identity | 42.28% | 48.66% | 56.92% |
| GMQE | 0.72 | 0.7 | 0.82 |
| QMEAN | -2.21 | -3.05 | 0.31 |
| Cβ | -2.48 | -3.18 | -0.66 |
| All Atom | -2.03 | -2.12 | -0.38 |
| Solvation | -1.65 | -1.91 | -0.37 |
| Torsion | -1.26 | -1.95 | 0.80 |

GMQE: Global Model Quality Estimation

QMEAN: Qualitative Model Energy ANalysis
